# *Peptoniphilus gorbachii* alleviates collagen-induced arthritis in mice by improving intestinal homeostasis and immune regulation

**DOI:** 10.3389/fimmu.2023.1286387

**Published:** 2024-01-04

**Authors:** Suhee Kim, Sung Hak Chun, Yun-Hong Cheon, Mingyo Kim, Hyun-Ok Kim, Hanna Lee, Seong-Tshool Hong, Sang-Jun Park, Myeong Soo Park, Young Sun Suh, Sang-Il Lee

**Affiliations:** ^1^ Department of Internal Medicine and Institute of Health Science, Gyeongsang National University School of Medicine and Hospital, Jinju, Republic of Korea; ^2^ Department of Internal Medicine, Gyeongsang National University Changwon Hospital, Changwon, Republic of Korea; ^3^ Department of Biomedical Sciences and Institute for Medical Science, Chonbuk National University Medical School, Jeonju, Republic of Korea; ^4^ Research Center, BIFIDO Co, Ltd, Hongcheon, Kangwon, Republic of Korea

**Keywords:** rheumatoid arthritis, microbiota, *Peptoniphilus gorbachii*, intestinal barrier integrity, inflammatory reaction

## Abstract

**Introduction:**

The intricate connection between gut microbiota and rheumatoid arthritis (RA) pathogenesis has gained prominence, although the specific microbial species contributing to RA development remain largely unknown. Recent studies have sought to comprehensively explore alterations in the human microbiome, focusing on identifying disease-related microbial species through blood analysis. Consequently, this study aimed to identify RA-associated microbial species using a serum microbial array system and to investigate the efficacy and underlying mechanisms of potential microbial species for RA treatment.

**Methods:**

Serum immunoglobulin M levels against 384 intestinal microbial species were assessed using a microbial microarray in patients with RA and healthy individuals. We investigated the therapeutic potential of the identified microbial candidate regarding arthritis development, immune responses, gut barrier function, and gut microbiome using a collagen-induced arthritis (CIA) mouse model.

**Results:**

Our findings revealed significant alterations in antibody levels against 36 microbial species in patients with RA compared to healthy individuals. Notably, the antibody levels against *Peptoniphilus gorbachii* (*PG*) were decreased in patients with RA and exhibited an inverse correlation with RA disease activity. *In vitro* experiments demonstrated that *PG* produced acetate and butyrate, while exhibiting anti-inflammatory properties. In CIA mice, *PG* administration suppressed arthritis symptoms, reduced the accumulation of inflammatory monocytes in the mesenteric lymph nodes, and downregulated gene expression of pro-inflammatory cytokines in the ileum. Additionally, *PG* supplementation restored intestinal barrier integrity and partially resolved gut microbial dysbiosis in CIA mice. The fecal microbiota in *PG*-treated mice corresponded to improved intestinal barrier integrity and reduced inflammatory responses.

**Conclusion:**

This study highlights the potential of serum-based detection of anti-microbial antibodies to identify microbial targets at the species level for RA treatment. Moreover, our findings suggest that *PG*, identified through the microbial microarray analysis, holds therapeutic potential for RA by restoring intestinal barrier integrity and suppressing the immunologic response associated with RA.

## 1 Introduction

Microbiota has emerged as a crucial factor in human health. Accumulating evidence demonstrates that dysbiosis, defined as imbalanced microbiota composition, is evident in patients with rheumatoid arthritis (RA) (1–5). A balanced microbiota and commensal microbe-derived metabolites are pivotal for maintaining symbiotic relationships within the intestine by reinforcing the epithelial barrier and regulating the mucosal immune response (6). However, microbial dysbiosis can compromise epithelial barrier function, resulting in an influx of bacteria and their metabolites into the bloodstream, leading to increased exposure of immune cells to bacterial antigens and subsequent pro-inflammatory responses (7). Furthermore, the enterotoxin zonulin, secreted by intestinal epithelial cells following microbial stimulation, reduces the expression of intestinal tight junction proteins. This, in turn, induces damage to the gut barrier, T cell-mediated mucosal inflammation, and the transmigration of immune cells from the gut to the joints (8). Therefore, restoring microbiota homeostasis and intestinal barrier function emerges as a promising strategy for preventing or treating RA (9).

Several microorganisms, including *Porphyromonas gingivalis* and *Prevotella copri*, have been implicated in RA pathogenesis*. P. gingivalis* triggers and exacerbates RA by activating Th17 immune responses and encoding peptidyl arginine deiminase, which facilitates the generation of RA-related autoantigen citrullinated peptides (10, 11). *Prevotella copri* has been discovered in both the preclinical stage and in new-onset stages of untreated RA patients and has been shown to contribute to the development of the disease (12, 13). *Lactobacillus salivarius* is consistently enriched in patients with RA, particularly those with highly active RA (14). Conversely, another *Prevotella* species, *Prevotella histicola*, suppresses arthritis via mucosal immune regulation (15). Additionally, *Lactobacillus casei* significantly suppresses the induction of arthritis, restores gut microbiota, and reduces oxidative stress (16, 17). These findings underscore that even within the same genus, distinct species can exert different effects on RA. Therefore, identifying RA-related microorganisms at the species level is pivotal for discovering effective targets for RA treatment.

While 16S rRNA gene sequencing and shotgun metagenomic sequencing of fecal samples are commonly used for identifying RA-associated microorganisms (18), they present limitations. Experimental conditions, such as sequencing errors and genomic repeats, can impact the outcome of these methods (19, 20). Additionally, the fecal microbiome only partially represents the entire gastrointestinal tract microbiota and fails to represent the mouth and lungs microbiota, both implicated in RA pathogenesis (21, 22). Furthermore, the translocation of transient gut microbiota could elicit persistent systemic responses, detectable through a serum microbial antibody array but frequently missed by metagenomic sequencing of fecal samples (23).

As a solution to sequencing limitations, serological testing emerges as a viable alternative for identifying disease-related microorganisms. Clinical practice often employs serological tests to identify causative microorganisms in pneumonia, scrub typhus, and syphilis. Similarly, serological tests have recently been conducted to investigate RA-related microbiota using blood samples. For example, antibodies against *P. gingivalis* were highly observed in patients with RA compared to healthy controls and associated with the presence of anti-cyclic citrullinated peptide antibodies (ACPA) (24–27). Elevated serum antibody levels against *Prevotella copri* were also observed in individuals at risk of or diagnosed with RA (28). Another recent study, using blood samples, demonstrated altered microbial small RNA composition in plasma of patients with RA compared to controls (29). Such blood-based investigations offer the advantage of reflecting changes in microorganisms across various organs, including the mouth, lungs, and intestine, with minimal sample requirements (30).

This study aimed to identify species-level microbial candidates associated with RA through a serum microbial antibody microarray and to demonstrate the therapeutic effects and underlying mechanisms of these candidate RA-related microbial species in collagen-induced arthritis (CIA) mice.

## 2 Materials and methods

### 2.1 Patients

The study included 81 patients with RA and 50 healthy controls (HC) matched for age, sex, and race. Human serum samples were provided by the Gyeongsang National University Hospital (GNUH)-Korea Biobank, sampled in 2017 and 2018. This study was approved by the Institutional Review Board (permit No: GNUH 2017-08-015). RA disease activity was assessed using the Disease Activity Score in 28 joints (DAS28) with measurements of erythrocyte sedimentation rate (ESR) and C-Reactive Protein (CRP) (31, 32). Patient information is summarized in **Supplementary Table 1**.

### 2.2 Anti-microbial antibody microarray and analysis

Immunoglobulin M (IgM) antibody levels targeting specific intestinal microbial species were evaluated in serum samples (33, 34). Specifically, 384 species of intestinal microbes obtained from the Gut Microbiota Bank (https://www.gutmicrobiotabank.com) were grown in yeast casitone fatty acid (YCFA) medium (**Supplementary Table 2**) and homogenized using an automill disruptor (Cosmo Bio, Japan). Microbial lysates were arrayed onto a nylon membrane using a microarray spotter (CapitalBio, China) and fixed by air drying, followed by a subsequent incubation at 80°C for 30 min. The membrane was then blocked in Tris-buffered saline containing 0.1% Tween 20 and 5% nonfat dry milk at 37 °C for 30 min. Patient serum was added to the chips, followed by a 30-min incubation at 37°C. Subsequently, the chips were incubated with Alexa Fluor 647-conjugated goat anti-human IgM secondary antibodies (Thermo Fisher Scientific, Waltham, MA, USA) at 37°C for 30 min. The fluorescence intensity was quantified using a Luxscan (CapitalBio, China). Each experiment involved at least three technical replicates and the average intensities of each sample were normalized using global normalization and Z-transform methods. Microbial richness and evenness were assessed based on species richness and Shannon index (α-diversity of species). Principal coordinate analysis (PCoA) was performed to identify microbial compositional diversity (β-diversity of species). Linear discriminant analysis (LDA) effect size (LEfSe) was performed using the Galaxy framework (https://huttenhower.sph.harvard.edu/galaxy/) to identify bacterial taxa enriched in the serum of healthy individuals and patients with RA (*p* < 0.01, LDA score > 3.0). A heatmap of bacterial species with differential relative abundances was generated (*p* < 0.01, Z-ratio > 1.5). Furthermore, a co-occurrence network was constructed using the Cytoscape v.3.10.0 plugin CoNet (*p* < 0.05 adjusted using the Benjamini-Hochberg correction, correlation coefficient > 0.5 based on four correlation measures including Spearman, Pearson, Bray-Curtis, and Kullback-Leibler).

### 2.3 Bacterial strains and culture supernatants

*Peptoniphilus gorbachii* (*PG*) strain WAL 10418 (JBP9-00201) and *P. gingivalis* (JBP11-00401) obtained from the Gut Microbiota Bank were cultured anaerobically at 37°C in YCFA medium. Live *PG* pellets and culture supernatants were prepared by centrifugation of bacterial culture at 12,000 × *g* for 10 min. Additionally, live *PG* pellets were suspended in 10% skim milk in YCFA and freeze-dried. Heat-killed (HK)-*PG* was generated by incubating the bacterial culture at 60°C for 1 h.

### 2.4 *In-vitro* bacterial competition assay

*PG* and *P. gingivalis* were grown separately in YCFA medium until reaching an OD_600_ of 0.1. They were then mixed at a 1:1 ratio. Pure cultures of *PG* and *P. gingivalis* were also prepared as controls to assess competition. Co-cultures and single cultures were serially diluted 10-fold eight times after 0, 8, and 16 h of culture. These dilutions were then spread onto agar plates and incubated at 37°C for 48 h. All single colonies visualized on the agar plates were identified by light microscopy following Gram-staining.

### 2.5 Quantification of short chain fatty acids (SCFAs)

SCFAs in *PG*-cultured supernatants were quantified using gas chromatography/mass spectrometry (GC-2010 Plus, GCMS-TQ 8030, Shimazu, Tokyo, Japan).

### 2.6 Cell culture and cytokine stimulation

RAW 264.7, a murine macrophage cell line, was maintained in Dulbecco’s modified eagle’s medium supplemented with 10% fetal bovine serum, 100 U/mL penicillin, and 100 mg/mL streptomycin (GIBCO, Carlsbad, CA, USA). RAW 264.7 cells were seeded in a 24-well plate (1 × 10^5^ cells/well) and cultured for 24 h. Subsequently, cells were exposed to HK-*PG* (1 × 10^5^ cells/well) or *PG* supernatant (0.5%) for 20 h, followed by stimulation with LPS (1 µg/mL; O55:B5, Sigma-Aldrich, St. Louis, MO, USA) for 4 h. Total RNA was extracted from the cells to evaluate pro-inflammatory gene expression.

Mouse small intestinal lamina propria (siLP) cells were isolated as previously described (35). Single-cell suspensions of mesenteric lymph nodes (MLN) were prepared through mechanical grinding and filtering using a nylon mesh. Single-cell suspensions from siLP and MLN were seeded into a 96-well plate (5 × 10^5^ cells/well) and stimulated with monoclonal anti-CD3e (1 µg/mL; clone 145-2C11, BD Biosciences, San Jose, CA, USA) and anti-CD28 (1 µg/mL; clone 37.51, BD Biosciences) antibodies, with or without HK-*PG* (5 × 10^3^ cells/well) or *PG* supernatant (0.5%) for 24 h. Cell culture supernatants were used for the measurement of IFN-γ and IL-17A levels.

### 2.7 Animals and arthritis induction

Five-week-old male DBA1/J mice were purchased from Central Laboratory Animal Inc. (Seoul, Korea). All mice were acclimatized for two weeks under specific pathogen-free conditions and fed standard rodent chow and sterile water. Animal experiments were conducted in accordance with the guidelines of Gyeongsang National University (GNU) and approved by the Institutional Animal Care and Use Committee (IACUC approval ID: GNU-200724-M0046) in Korea.

CIA was induced as previously described (36). Briefly, mice were immunized intradermally at the base of the tail with 100 μg bovine type II collagen (CII, Chondrex, Redmond, WA, USA) emulsified with complete Freund’s adjuvant (CFA, Sigma-Aldrich) on day 0. Three weeks later (day 21), mice were boosted with CII emulsified with incomplete Freund’s adjuvant (IFA, Chondrex). Mice were divided into four groups according to the treatment scheme: (1) naïve mice without treatment and immunization (*n* = 4), (2) CII-immunized mice without treatment (CIA, *n* = 8), (3) *PG*-treated CIA mice (CIA/*PG*, *n* = 9), and (4) antibiotic-treated CIA mice (CIA/AB, *n* = 8). *PG* treatment was initiated four weeks before the first CII injection and continued for 9 weeks until the end of the experiment (day 35). A total of 1 × 10^9^ colony-forming units (CFUs) of live *PG* suspended in 200 µL of PBS were orally administered to mice three times a week. For antibiotic treatment, ampicillin (1 g/L), vancomycin (0.5 g/L), neomycin (1 g/L), and metronidazole (1 g/L) were administered to mice in the drinking water for a total period of 3 weeks, beginning on day 14 after the first CII injection.

Clinical arthritis scores were evaluated on a scale of 0–4 for each limb three times a week, starting after the second CII injection (day 21), as previously described (36). Mice were sacrificed five weeks post-immunization (day 35) for sample collection.

### 2.8 Histopathological examination

Ankles were formalin-fixed, decalcified with a decalcifying solution (Sigma-Aldrich), and embedded in paraffin. Ankle joint sections (5 μm) were subjected to hematoxylin and eosin (H&E) and safranin O staining. Slides were imaged using a Nikon microscope imaging system and NIS-Elements software (Nikon Instruments, Tokyo, Japan). The assessment of synovial inflammation, bone erosion, and cartilage damage was conducted as previously described (36).

### 2.9 CFU assay for *P. gorbachii* colonization in feces

Freshly collected mouse fecal pellets were smashed in 1 mL of YCFA medium, followed by a 10-fold serial dilution six times. One hundred microliters of the serially diluted fecal suspension were spread onto YCFA agar medium, and the agar plates were incubated at 37°C for 16 h in anaerobic conditions. Following visualization of single colonies on the agar plate, approximately 100 single colonies were selected from the plates. The 16S rDNA sequences, encompassing regions V1-V9, were amplified using colony PCR and universal primers (27F 5’-AGAGTTTGATCCTGGCTCAG-3’ and 1492R 5’-GGTTACCTTGTTACGACTT-3’). These amplified DNA fragments were sequenced using Sanger sequencing. Full-length 16S rRNA sequences were analyzed in a DNAbaser4.36 analyzer, and contigs of each individual DNA were uploaded to NCBI BLAST for bacterial species identification.

### 2.10 Quantitative real-time PCR

Total RNA was extracted using TRIzol reagent (Thermo Fisher Scientific) and reverse transcribed using an iScript cDNA synthesis kit (Bio-Rad, Hercules, CA, USA). DNA was subjected to qPCR amplification using a SYBR Green PCR master mix (Thermo Fisher Scientific) on a ViiA 7 Real-time PCR Detection System (Applied Biosystems, Waltham, MA, USA). The expression of target genes relative to glyceraldehyde 3-phosphate dehydrogenase was calculated using the 2^-ΔΔCt^ comparative method. The primer pairs used are listed in **Supplementary Table 3**.

### 2.11 Enzyme-linked immunosorbent assay (ELISA)

To measure IFN-γ and IL-17A levels in cell culture supernatants, 96-well plates (Greiner Bio-One GmbH, Frickenhausen, Germany) were coated with IFN-γ (eBioscience, San Diego, CA, USA) or IL-17A monoclonal antibodies (eBioscience) and blocked with BD OptEIA™ Assay diluent (BD Biosciences). Samples were added to the wells and incubated overnight at 4°C. Biotinylated IFN-γ (eBioscience) or IL-17A (eBioscience) antibodies were added, followed by the addition of AKP streptavidin (BD Biosciences). Color was developed by adding 5 mM phosphatase substrate (Sigma-Aldrich), and absorbance was measured at 405 nm using a Versamax microplate reader with SoftMax Pro 6.5.1 software (Molecular Devices, Wokingham, UK).

Serum samples were prepared by centrifuging whole blood collected from mice at 2,000 × *g* for 10 min at 4°C. Subsequently, the serum samples were subjected to dilutions of 100,000-fold, 4-fold, and 1000-fold for the detection of anti-CII IgG antibody (Chondrex), anti-CCP antibody (MyBioSource Inc., San Diego, CA, USA), and zonulin (MyBioSource Inc.), respectively. The levels of serum autoantibodies and zonulin (MyBioSource Inc.) were measured in accordance with the manufacturer’s instructions.

### 2.12 Flow cytometric analysis

Mouse colonic lamina propria (cLP) cells were isolated as previously described (35), and MLN and ILN cell suspensions were prepared through mechanical grinding. Cell surface and intracellular cytokine staining were conducted as previously described (35). Cells stained with fluorochrome-conjugated antibodies (**Supplementary Table 4**) were analyzed using a LSRFortessaTM X-20 flow cytometer (BD Biosciences). Data analysis was carried out using FlowJo (Tree Star Inc., Ashland, OR, USA).

### 2.13 16S rRNA gene sequencing and analysis

Total bacterial genomic DNA from intracecal samples was extracted using the MagMAX™ Microbiome Ultra Nucleic Acid Isolation Kit on a KingFisher Flex automated DNA/RNA isolation system (Thermo Fisher Scientific), according to the manufacturer’s instructions. DNA samples were analyzed using a Nano-300 Micro-spectrophotometer (ALLSHENG, Hangzhou, China) and Qubit 3.0 fluorometer (Thermo Fisher Scientific) to assess concentration and purity of fecal DNA.

Libraries were constructed using the 16S Metagenomic Sequencing Library Preparation guide protocol (Illumina, San Diego, CA, USA). Variable regions 3 and 4 of the bacterial 16S rRNA gene were amplified using Kapa HiFi Hotstart Ready PCR Mix (Kapa Biosystems, Wilmington, MA, USA), with primers targeting MiSeq 341F and 805R. DNA indexing and second PCR were performed using Nextera XT index kits (Illumina). Each PCR product was purified using an Agencourt AMPure XP purification system (Beckman Coulter, Brea, CA, USA). Quantification and size estimation of the library were performed using the QIAxcel Advanced system and QIAxcel DNA High Resolution Kit (Qiagen, Hilden, Germany). Amplicons were pooled for sequencing using an Illumina MiSeq System (2 × 300 bp paired-end reads).

Sequence assembly and quality filtering were processed using QIIME2 software. Raw sequence reads were demultiplexed using the q2-demux plugin, followed by denoising using the DADA2 plugin to eliminate low-quality scores. All amplicon sequence variants (ASVs) were classified using the SILVA 138 99% database. QIIME2’s diversity analyses (q2-diversity plugin) and R software were used to present microbial richness, evenness diversity indices, and PCoA. The LEfSe algorithm was implemented using the Galaxy computational tool.

### 2.14 Statistical analysis

Data visualization and analysis were performed using GraphPad Prism (GraphPad, San Diego, CA, USA) or IBM SPSS (SPSS Inc., Chicago, IL, USA). Statistical significance was evaluated using a t-test or one- or two-way analysis of variance (ANOVA) followed by Fisher’s LSD *post hoc* test, or a Mann-Whitney test, Wilcoxon test or Kruskal-Wallis test followed by Dunn’s *post hoc* test, depending on the normal distribution of the data. Pearson’s correlation was used to assess the correlation between relative bacterial abundances and variables. Permutational multivariate analysis of variance (PERMANOVA) was applied using the QIIME2 plugin to evaluate differences in microbial community structure. Unless otherwise indicated, data are presented as the mean ± standard error of the mean (SEM), and a *p* value lower than 0.05 was considered significant.

## 3 Results

### 3.1 Perturbation of serum anti-microbial antibodies in RA patients

**Figure 1:**
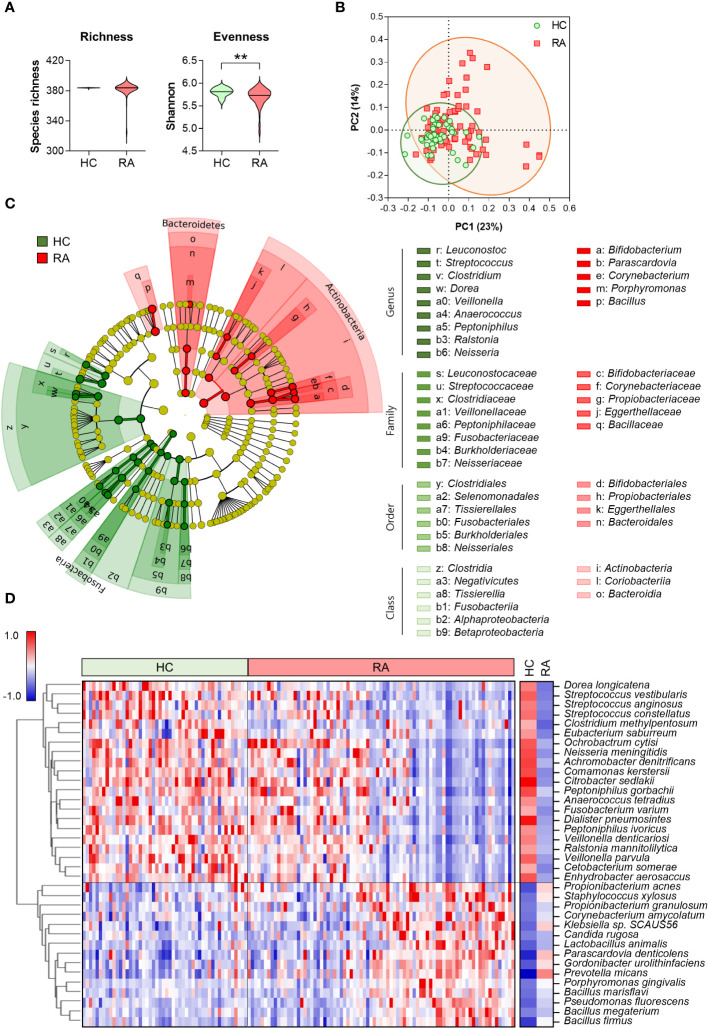
Serum anti-microbial antibody profile in healthy control (*n* = 50) and patients with RA (*n* = 81). **(A)** α-diversity assessed through microbial species-specific antibody abundance richness and evenness (Mann-Whitney test, **p < 0.01). **(B)** β-diversity analyzed using Bray-Curtis PCoA of microbial species-specific antibody communities (PERMANOVA, *p* < 0.001 vs HC). Symbols represent each individual. **(C)** Cladogram generated following a LEfSe analysis at the phylum to genus level (pairwise Wilcoxon test, *p* < 0.01, LDA score > 3.0). Green: taxa enriched in HC, Red: taxa enriched in RA. **(D)** Heatmap depicting differential anti-microbial antibody abundances between HC and patients with RA (t- and z-tests, *p* < 0.01, Z-ratio > 1.5). Blue: low abundance, Red: high abundance. HC: Healthy control, RA: Rheumatoid arthritis, PCoA: Principal coordinates analysis, LDA: Linear discriminant analysis, LEfSe: LDA effect size.

To investigate microbial alterations in patients with RA based on serum antibody profiling, we conducted microbial species-specific IgM microarrays. The α-diversity of serum anti-microbial antibodies revealed a significant decrease in evenness in patients with RA compared to HC, while the richness remained similar between the two groups (**Figure 1A**). This diversity was further evident in the β-diversity assessment, where PCoA highlighted distinct clustering patterns representing the varying composition of serum anti-microbial antibodies in RA patients and HC (**Figure 1B**).

We identified discriminative core microbes employing a bacterial taxonomic rank based on the relative abundance of anti-microbial antibodies in the serum from HC and RA patients using an LEfSe analysis (**Figure 1C**). At the phylum level, Fusobacteria was significantly enriched in HC, whereas Actinobacteria and Bacteroidetes were enriched in patients with RA. At low taxonomic ranks, 6 and 3 classes, 6 and 4 orders, and 8 and 5 families were enriched in HC and patients with RA, respectively. The 9 genera including *Streptococcus*, *Clostridium*, and *Peptoniphilus*, were significantly enriched in HC, whereas 5 genera, including *Corynebacterium*, *Prophyromonas*, and *Bacillus*, were significantly enriched in patients with RA.

The heatmap depicting serum microbial species-specific antibody abundances highlighted significant alterations in antibody abundance against 36 microbial species in patients with RA compared to HC (**Figure 1D**). Specifically, antibodies against 21 microbial species were decreased, while antibodies against 15 microbial species were increased in patients with RA compared to HC.

### 3.2 Altered serum anti-microbial antibodies are associated with disease severity in patients with RA

**Figure 2:**
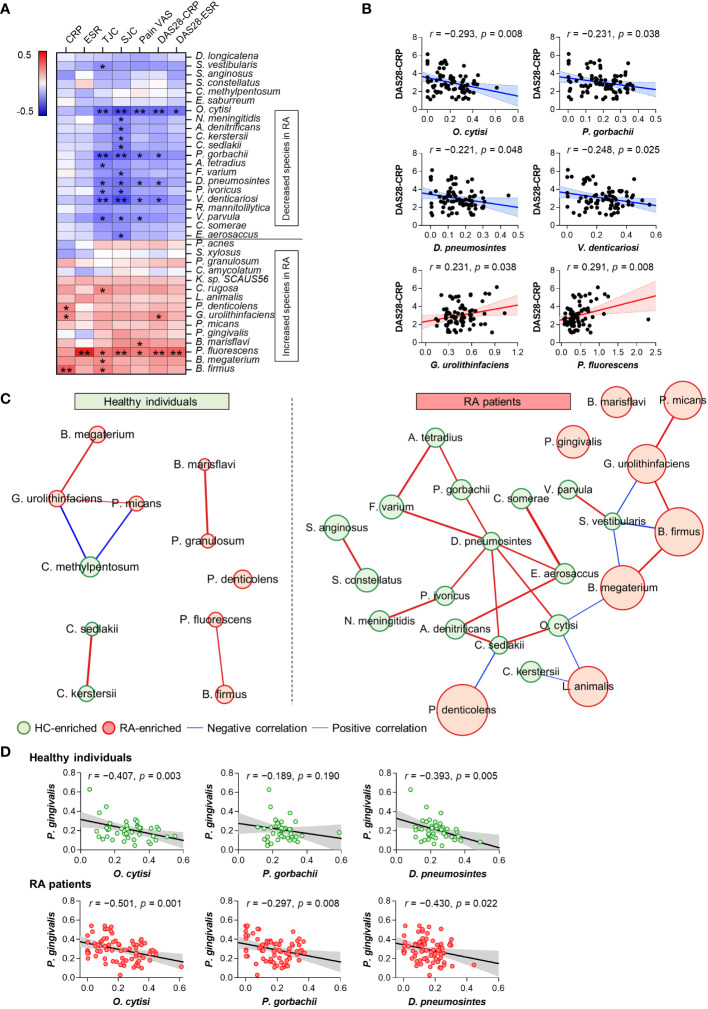
Association between altered microbial species-specific antibodies and disease activity in patients with RA (*n* = 81). **(A)** Correlations between differential anti-microbial antibody abundances and clinical features in patients with RA (Pearson’s correlation, *p <0.05, **p < 0.01). Blue: inverse correlation, Red: positive correlation. **(B)** The linear relationships between differential anti-microbial antibody abundances and DAS28 score in patients with RA (Pearson’s correlation). Solid lines represent the mean linear regression, while dotted lines indicate 95% confidence intervals. Symbols represent individual RA patients. **(C)** Co-occurrence network of differential anti-microbial antibodies in HC (*n* = 50) and patients with RA (*n* = 81). The size of each circle (node) represents the abundance of anti-microbial antibodies enriched in either HC (green node) or RA (red node) patients (Benjamini-Hochberg’s correction, *p* < 0.01). Lines connecting two nodes (edges) indicate the correlation efficiency between anti-microbial antibodies (chosen by Spearman, Pearson, Bray-Curtis, and Kullback-Leibler measures, *r* > 0.5, *p* < 0.05). Blue edges: negative correlation, Red edges: positive correlation. **(D)** The linear relationships between antibodies against *P. gingivalis* and disease activity-inversely correlated microbial species in HC and patients with RA (Pearson’s correlation). Solid lines represent the mean linear regression, while gray-filled areas indicate 95% confidence intervals. Symbols represent each individual. ESR: Erythrocyte Sedimentation Rate, CRP: C-Reactive Protein, TJC: Tender Joint Count, SJC: Swollen Joint Count, VAS: Visual Analog Scale, DAS: Disease Activity Score.

Our study identified significant alterations in 36 anti-microbial antibodies in the comparison of patients with RA and HC (**Figure 1D**). Correlation analysis was conducted to investigate the association between the abundances of these significant anti-microbial antibodies and RA disease activity, determining associations with disease severity. Notably, anti-microbial antibodies decreased in patients with RA were inversely correlated with disease activity, whereas those elevated in patients were positively correlated with disease activity (**Figure 2A**). Specifically, the antibodies against *Ochrobactrum cytisi* (*O*. *cytisi*), *P. gorbachii*, *Dialister pneumosintes* (*D*. *pneumosintes*), and *Veillonella denticariosi* (*V*. *denticariosi*) were associated with lower disease severity (**Figure 2B**). Conversely, antibodies against *Gordonibacter urolithinfaciens* (*G*. *urolithinfaciens*) and *Pseudomonas fluorescens* (*P. fluorescens*) were associated with higher disease severity, as assessed by DAS28-CRP in patients with RA.

The CoNet approach was employed to infer association patterns among significant 36 anti-microbial antibodies in patients with RA and HC, revealing a higher number of networks in patients with RA compared to HC (**Figure 2C**), which is suggestive of increased co-occurrences of anti-microbial antibodies in patients with RA. In the RA patient group, most of the networks exhibited direct or indirect connections to one another. Specifically, antibodies against *O*. *cytisi*, *P. gorbachii*, and *D*. *pneumosintes*, which were enriched in HC and inversely associated with disease activity (**Figure 2B**), demonstrated positive connections either directly or indirectly within the RA patient network (**Figure 2C**). Additionally, the anti-*G. urolithinfaciens* antibody, enriched in RA and positively associated with disease activity (**Figure 2B**), showed positive connections with RA-enriched antibodies such as *P. micans* and *B. firmus*, while displaying an inverse connection with the HC-enriched *S. vestibularis* antibody. Overall, the altered anti-microbial antibodies exhibited associations with disease severity and interconnected relationships (networks) among themselves in patients with RA.

The analysis of microbial arrays revealed an increased abundance of antibodies targeting *P. gingivalis* in patients with RA, a putative pathogen implicated in RA through protein citrullination (**Figure 1D**). To further identify specific microbial candidates for RA therapy, we investigated the relationship between antibodies against *P. gingivalis* and three microbial species: *O*. *cytisi*, *P. gorbachii*, and *D*. *pneumosintes*. These species were selected based on their association with disease severity and their co-occurrence network (**Figures 2B, C**). The abundance of anti-*O*. *cytisi* and anti-*D*. *pneumosintes* antibodies exhibited an inverse correlation with the abundance of anti-*P. gingivalis* antibody in both patients with RA and HC (**Figure 2D**). Notably, the abundance of anti-*P. gorbachii* antibody showed an inverse correlation with anti-*P. gingivalis* antibody solely among patients with RA (**Figure 2D**). Importantly, *P. gorbachii* (*PG*) has been reported to inhibit infection (37) and produce SCFAs which were associated with RA suppression (38). Therefore, based on our bacterial microarray findings and the reported evidence (**Supplementary Table 5**), we selected *PG* as a potential specific target for RA treatment and further explored its involvement in RA.

### 3.3 *In-vitro* characteristics of *P. gorbachii*

**Figure 3:**
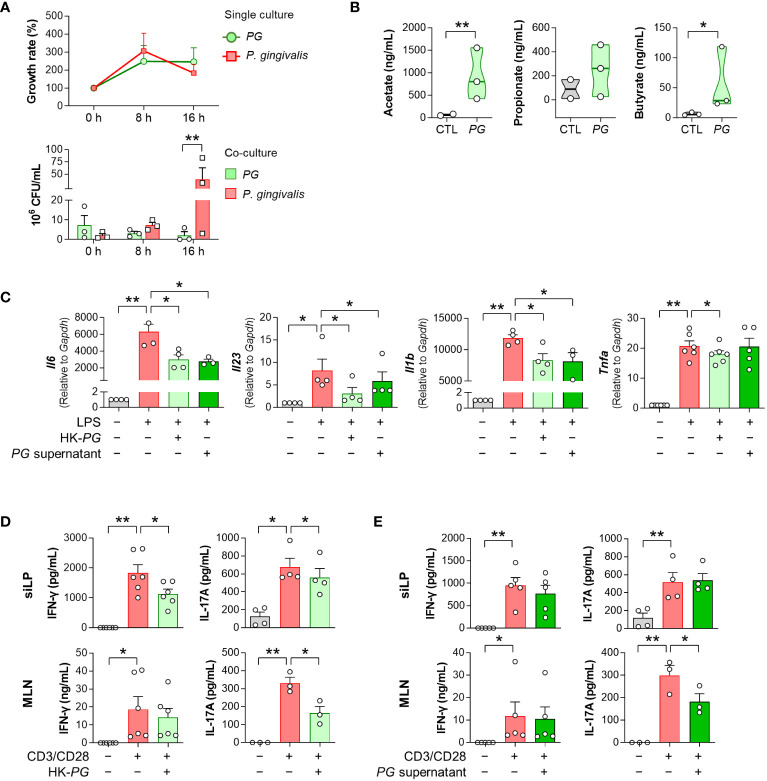
*In vitro* physiological and immunological properties of *P. gorbachii* (*PG*). **(A)** *In vitro* competition assay between *PG* and *P. gingivalis*. *PG* and *P. gingivalis* cultures were prepared each as a control (single culture) and then mixed (co-culture). After 0, 8, and 16 h of single and co-cultures, *PG* and *P. gingivalis* colonies were counted. *n* = 3 for each group and time (Two-way ANOVA, Fisher’s LSD *post hoc* test, **p < 0.01). **(B)** Measurement of short-chain fatty acids (SCFAs) in *PG*-cultured supernatants. *n* = 2–3 per group (t-test, *p < 0.05, **p < 0.01). Median and interquartile range (1^st^ – 3^rd^ quartile). **(C)** Pro-inflammatory gene expression of RAW 264.7 cells cultured in the absence or presence of heat-killed (HK)-*PG* or *PG* supernatant under LPS stimulation. *n* = 3–6 per group (One-way ANOVA, Fisher’s LSD *post hoc* test, *p < 0.05, **p < 0.01 vs LPS^+^ control). **(D, E)** IFN-γ and IL-17A concentrations in mouse siLP and MLN cell supernatants cultured in the absence or presence of HK-*PG* **(D)** or *PG* supernatant **(E)** under anti-CD3e and anti-CD28 stimulation. *n* = 3–6 per group (One-way ANOVA, Fisher’s LSD *post hoc* test, *p < 0.05, **p < 0.01 vs CD3/CD28^+^ control). Mean ± SEM. siLP: Small intestinal lamina propria, MLN: Mesenteric lymph node.

To investigate the potential mechanisms underlying the therapeutic effects of *PG* in RA, we examined the competitive potential of *PG* against *P. gingivalis*, an RA-associated pathogen. Our *in-vitro* interspecies competition assay revealed an inhibitory impact of *P. gingivalis* on *PG* (**Figure 3A**). Despite similar growth rates in single cultures of both species, the growth of *PG* was strongly suppressed after 16 h of co-culture with *P. gingivalis*, indicating interference in bacterial growth between the two species.

To validate the ability of *PG* to produce SCFAs, which have therapeutic implications in RA, we performed GC-MS analysis of *PG*-cultured media. *PG* produced SCFAs, including acetate and butyrate (**Figure 3B**), which have been associated with the alleviation of RA symptoms. Subsequently, we assessed the ability of *PG* to suppress immune responses through evaluation of pro-inflammatory cytokine profiles in RAW 264.7 macrophages, siLP, and MLN cells treated with HK-*PG* or *PG* supernatants under cell-stimulating conditions. Both HK-*PG* and *PG* supernatant downregulated the gene expression of LPS-induced *Il6*, *Il23*, *Il1b*, and *Tnfa* in RAW 264.7 cells (**Figure 3C**; **Supplementary Figure 1**). Moreover, HK-*PG* significantly reduced CD3/CD28-induced IFN-γ and IL-17A production in siLP cells, as well as IL-17A production in MLN cells (**Figure 3D**). However, *PG* supernatant only reduced CD3/CD28-induced IL-17A production in MLN cells (**Figure 3E**), indicating a specific anti-inflammatory suppression of immune cells. In an additional *in-vitro* experiment involving *P. fluorescens*, *P. gingivalis*, and *P. micans*, bacteria identified as elevated in the serum bacterial microarray analysis of patients with RA, treatment with *P. fluorescens* and *P. gingivalis* independently led to the upregulation of pro-inflammatory cytokine genes in RAW 264.7 cells in the absence of LPS (**Supplementary Figure 2A**). Meanwhile, the treatment of *P. gingivalis* and *P. micans* did not impact the induction of pro-inflammatory cytokine genes under LPS stimulated condition (**Supplementary Figure 2B**). Overall, *PG* demonstrated an outstanding effect in inhibiting the upregulation and secretion of pro-inflammatory cytokines associated with activated macrophages and CD4^+^ T cells. These findings underscore the critical role of *PG* in the modulating both innate and adaptive immune responses, involving key immune cell types such as macrophages and CD4^+^ T cells, which play a central role in the pathogenesis of RA.

### 3.4 *P. gorbachii* treatment suppresses arthritis in CIA mice

**Figure 4:**
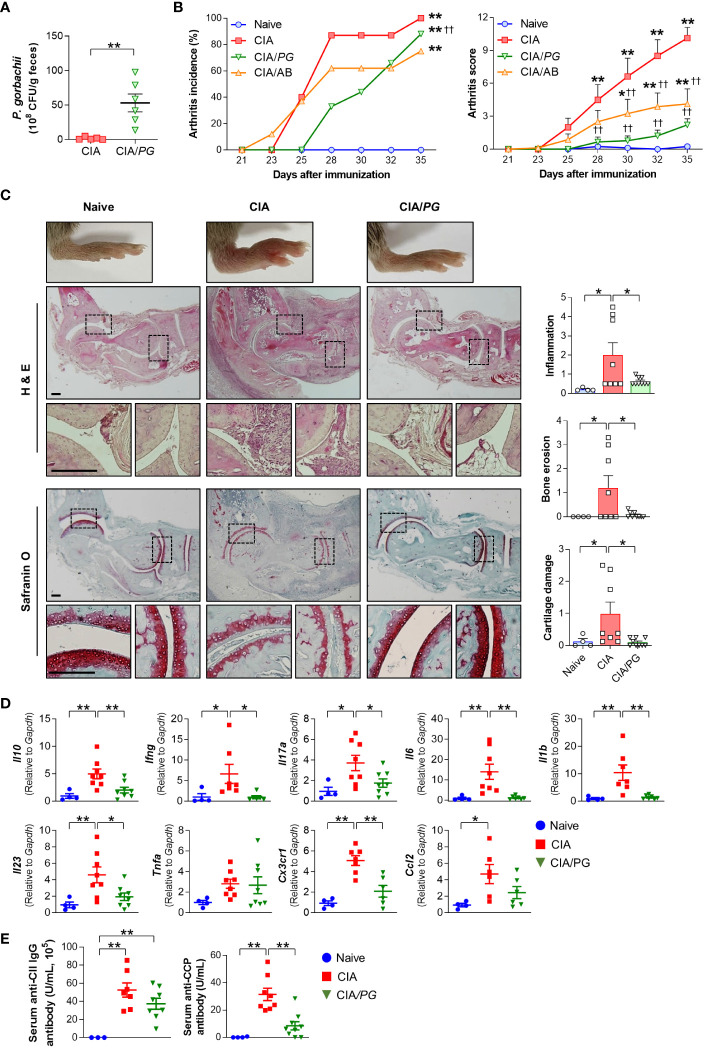
Effect of *PG* on arthritis in CIA mice. CIA was induced by intra-dermal injection of CII into mice. *PG* was orally administered for 4 weeks before the first CII injection (day 0) and continued until euthanasia at day 35. Samples were collected from naïve (*n* = 4), CIA (*n* = 8), and CIA/*PG* (*n* = 9) mice at day 35. **(A)** Colonization of *PG* in feces from CIA and CIA/*PG* mice (t-test, **p < 0.01). **(B)** Arthritis incidence and scores in the experimental groups (Log-rank test and Two-way ANOVA, Fisher’s LSD *post hoc* test, *p < 0.05, **p < 0.01 vs naïve; ^††^ *p* < 0.01 vs CIA). **(C)** Representative histopathological images and scores of ankle joints stained with H&E and safranin O (One-way ANOVA, Fisher’s LSD *post hoc* test, *p < 0.05). Scale bar: 200 μm. **(D)** Expression of anti- and pro-inflammatory cytokine genes in ankle samples (One-way ANOVA, Fisher’s LSD *post hoc* test, *p < 0.05, **p < 0.01). **(E)** Serum anti-CII IgG and anti-CCP antibody levels (One-way ANOVA, Fisher’s LSD *post hoc* test, **p < 0.01). Mean ± SEM.

To determine whether *PG* can prevent RA development *in vivo*, we evaluated the effect of *PG* treatment on arthritis in a CIA mouse model. First, we compared fecal CFUs of *PG* between CIA and *PG*-fed CIA (CIA/*PG*) mice to determine colonization of the intestine following oral administration. The fecal CFUs of *PG* were significantly higher in CIA/*PG* mice than in CIA mice, indicating stable intestinal colonization (**Figure 4A**).

To clarify the effect of *PG* on arthritis, we treated a group of mice with antibiotics, as a broad spectrum of antibiotics has been reported to reduce arthritis severity in CIA mice (39). Both antibiotic- (CIA/AB) and *PG*-treated (CIA/*PG*) CIA mice exhibited reduced arthritis scores compared to CIA mice (**Figure 4B**). Furthermore, CIA/*PG* mice showed a clear prevention in arthritis development, with a lower incidence of arthritis than that of CIA mice (**Figure 4B**). Histopathological analysis using H&E staining revealed decreased inflammatory cell infiltration and bone erosion in CIA/*PG* mice (**Figure 4C**). Additionally, PG treatment led to the recovery of cartilage thinning in the joints of CIA mice.

To further confirm the suppressive effect of *PG* on arthritis, we investigated the gene expression of pro-inflammatory cytokines and chemokines in the ankles of the experimental groups. CIA/*PG* mice exhibited downregulated gene expression of pro-inflammatory cytokines (*Ifng*, *Il17a*, *Il6*, *Il1b*, and *Il23*) and a chemokine receptor (*Cx3cr1*), which were upregulated in CIA mice (**Figure 4D**; **Supplementary Figure 3A**). The gene expression of the anti-inflammatory cytokine *Il10* was also upregulated in the ankles of CIA mice and normalized by *PG* treatment (**Figure 4D**).

Next, we assessed whether *PG* treatment influenced antigen-specific systemic autoimmunity by measuring serum anti-CII IgG and anti-CCP antibody levels. We observed no significant difference in anti-CII IgG antibody levels between CIA and CIA/*PG* mice; however, CIA/*PG* mice exhibited a trend for lower levels of anti-CII IgG antibody compared to CIA mice (**Figure 4E**). Furthermore, the elevated serum anti-CCP antibody levels in CIA mice were significantly decreased with *PG* treatment (**Figure 4E**). These findings suggest that *PG* not only attenuates arthritis severity but also reduces the production of antigen-specific autoantibodies, reflecting its potential therapeutic role in RA.

### 3.5 *P. gorbachii* is involved in the suppression of arthritis by improving intestinal inflammation and barrier integrity in CIA mice

In our study, we explored *PG*’s role in regulating immune responses in the gut-joint axis, as prior research has suggested that intestinal inflammation can trigger the onset of RA (40). We conducted an analysis of T cell subsets in the ILN, MLN, and cLP of naïve, CIA, and CIA/*PG* mice using flow cytometry. The detailed gating strategies can be found in **Supplementary Figure 4A**.

**Figure 5:**
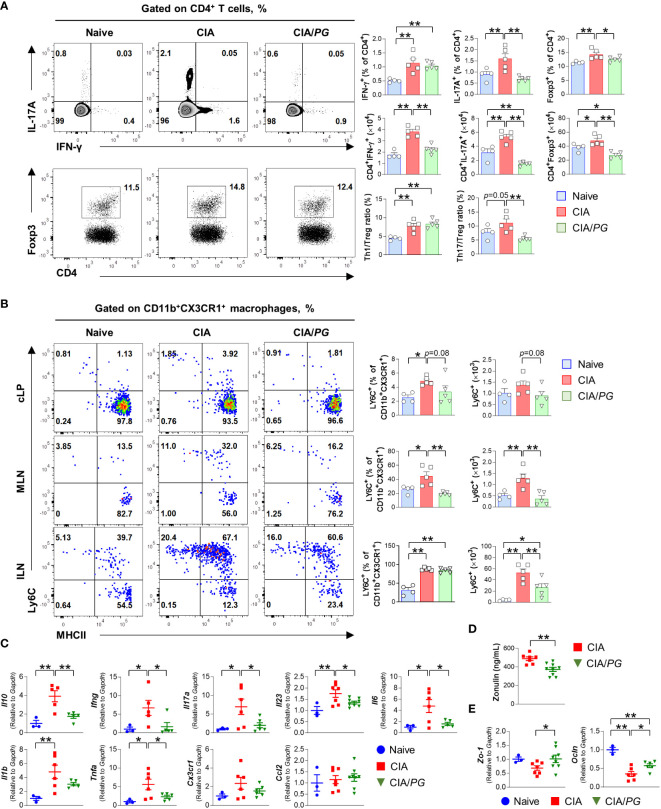
Role of *PG* in CIA-characteristic inflammation and intestinal barrier function. Samples were collected from naïve, CIA, and CIA/*PG* mice at 35 days after CII immunization. **(A, B)** Flow cytometry analysis of T cell subsets in the ILN **(A)** and macrophage lineage cells **(B)** from the cLP, MLN, and ILN of naïve (*n* = 4), CIA (*n* = 5), and CIA/*PG* (*n* = 5) mice. **(C–E)** Gene expression of pro-inflammatory cytokines in ileum **(C)**, zonulin levels in serum **(D)**, and gene expression of *Zo-1* and *Ocln* in ileum **(E)** of naïve (*n* = 3), CIA (*n* = 6–7), and CIA/*PG* (*n* = 6–9) mice. Mean ± SEM. One-way ANOVA, Fisher’s LSD *post hoc* test **(A–C, E)** and t-test **(D)**, *p < 0.05, **p < 0.01. cLP: Colonic intestinal lamina propria.

Notably, we observed a significant decrease in the percentage of Th17 (CD4^+^IL-17A^+^) cells in the ILN of CIA/*PG* mice compared with that in CIA mice (**Figure 5A**). Furthermore, *PG* treatment significantly diminished the numbers of Th1 (CD4^+^IFN-γ^+^) and Th17 cells that had accumulated in the ILN of CIA mice. Interestingly, Treg (CD4^+^Foxp3^+^) cells, well-known for their immunosuppressive properties, were also increased in CIA mice relative to naïve and CIA/*PG* mice. However, the Th1/Treg and Th17/Treg ratios were higher in CIA mice than in naïve mice, suggesting a preferential differentiation or proliferation toward Th1 and Th17 cells in CIA. Importantly, *PG* treatment effectively restored the Th17/Treg imbalance observed in CIA mice by decreasing the Th17/Treg ratio in CIA/*PG* mice compared to CIA mice.

Although no difference was observed in Th1 and Th17 cells of cLP and MLN between naïve, CIA, and CIA/*PG* mice (**Supplementary Figure 4B**), *PG* treatment significantly reduced the accumulation of inflammatory monocytes (CD11b^+^CX3CR1^+^Ly6C^+^) in the MLN and ILN of CIA mice (**Figure 5B**). Furthermore, our study encompassed an analysis of conventional dendritic cells (cDCs), with a specific focus on the CD103^–^ cDC2 subset, known for its role in promoting T cell differentiation into Th17 cells. However, our investigation did not reveal significant differences in CD103^–^ cDC2 subset within the cLP and MLN when comparing non-treated CIA and *PG*-treated CIA mouse groups (**Supplementary Figure 4C**). Although *PG* treatment led to a reduction in both the number and percentage of CD103^–^ cDC2 cells in the ILN of CIA mice, it did not restore normalcy to the population of all cDC subsets within cLP and MLN (**Supplementary Figure 4C**). Collectively, our findings highlight the potent inhibitory effect of *PG* on Th17 cells in ILN and the marked reduction in the accumulation of inflammatory monocytes in both ILN and gut-associated MLN.

To further understand the intestinal effect of *PG* on RA pathogenesis, we investigated the gene expression of pro-inflammatory cytokines in the ileum of naïve, CIA, and CIA/*PG* mice. The gene expression of *Ifng*, *Il17a*, *Il23*, *Il6*, and *Tnfa* was decreased in the ileal tissues of CIA/*PG* mice compared to those in CIA mice (**Figure 5C**). Moreover, we evaluated gut barrier integrity using zonulin, which is a protein secreted from gut epithelial cells in response to dietary, gut microbial, or inflammatory stimuli (8). Serum zonulin levels were decreased in CIA/*PG* mice compared to CIA mice, indicating improved gut barrier integrity (**Figure 5D**). Furthermore, genes encoding tight-junction proteins, *zonula occludens-1* (*Zo-1*) and *occludin* (*Ocln*), were upregulated in the ileum of CIA/*PG* mice compared to those in CIA mice (**Figure 5E**; **Supplementary Figure 3B**). These data suggest a protective capacity of *PG* against arthritis through inhibition of CIA intestinal inflammation, reflected in the prevention of increased gut permeability and the disassembly of tight junctions.

### 3.6 *P. gorbachii* treatment restores gut microbial alterations in CIA mice

**Figure 6:**
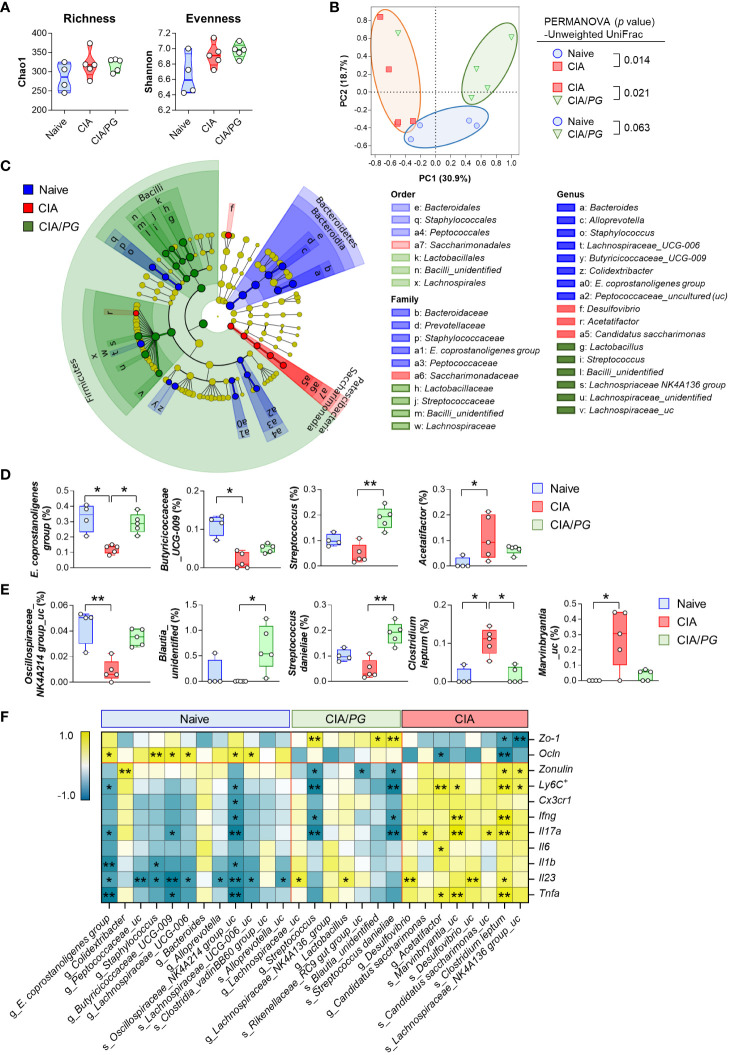
Role of *PG* in gut microbiome in CIA. Cecal samples were collected from naïve (*n* = 4), CIA (*n* = 5), and CIA/*PG* (*n* = 5) mice at 35 days after CII immunization and 16S rRNA gene sequencing was conducted. **(A)** α-diversity measured based on the Chao1 and Shannon indices (One-way ANOVA, Fisher’s LSD *post hoc* test, *p* > 0.05). Median and interquartile range (1^st^ – 3^rd^ quartile). **(B)** β-diversity assessed using PCoA based on unweighted UniFrac distance matrix. **(C)** Cladogram constructed based on the LEfSe analysis. Colors indicate bacterial taxa discriminately enriched in cecal microbiome of naive (blue), CIA (red), and CIA/*PG* (green) mice (Kruskal-Wallis, pairwise Wilcoxon test, *p* < 0.05, LDA score > 2.0). **(D, E)** Relative abundance of significantly different genera **(D)** and species **(E)** between groups (Kruskal-Wallis, Dunn’s *post hoc* test). Median ± min to max. **(F)** Relationship between gut-associated inflammatory profiles and cecal microbial taxa discriminately enriched in each experimental group (Pearson’s correlation, *p < 0.05, **p < 0.01). Colors indicate negative (turquoise) and positive (yellow) correlations.

To investigate the effects of *PG* treatment on the gut microbiome, we conducted 16S rRNA gene sequencing analysis of cecal samples from naïve, CIA, and CIA/*PG* mice. Although no significant differences were observed in α-diversity using Chao1 and Shannon indices among cecal microbiota from naïve, CIA, and CIA/*PG* mice (**Figure 6A**), CIA promoted a change in microbial communities with a clear separation in PCoA plots from the naïve and CIA/*PG* groups (**Figure 6B**). The cladogram showed discriminative bacterial clades by comparison of different mouse groups, where Bacteroidetes, Patescibacteria, and Firmicutes were the microbial phyla with differential abundances in the cecum of naïve, CIA, and CIA/*PG* mice, respectively (**Figure 6C**). At lower taxonomic ranks, bacterial taxa enriched in the cecum of naïve mice were *Bacteroidaceae*, *Prevotellaceae*, *Staphylococcaceae*, *Eubacterium coprostanoligenes* (*E*. *coprostanoligenes*) group, and *Peptococcaceae* families, as well as eight genera including *Bacteroides*, *Alloprevotella*, *Staphylococcus*, *Lachnospiraceae_UCG-006*, *Butyricicoccaceae_UCG-009*, and *E*. *coprostanoligenes* group. Bacterial taxa enriched in the cecum of CIA mice were the *Saccharimonadaceae* family, as well as the *Desulfovibrio*, *Acetatifactor*, and *Candidatus saccharimonas* genera. Bacterial taxa enriched in the cecum of CIA/*PG* mice were *Lactobacillaceae*, *Streptococcaceae*, *Bacilli_unidentified*, and *Lachnospiraceae* families, as well as six genera including *Lactobacillus*, *Streptococcus*, and *Lachnospriaceae NK4A136* group.

*PG* treatment restored certain microbial genera and species to levels similar to those of naïve mice (**Figures 6D, E**). Notably, the relative abundance of the *E*. *coprostanoligenes* group and *Clostridium leptum* (*C. leptum*) was significantly normalized in the cecum of CIA/*PG* mice. In addition, *PG* treatment also promoted a significant increase in *Blautia_unidentified* and *Streptococcus danieliae* (*S. danieliae*) compared to CIA mice (**Figure 6E**). Overall, *PG* treatment partially resolved CIA-induced gut microbial dysbiosis, bring the gut microbial profile closer to that of naïve mice. To explore whether these changes in gut microbiota induced by *PG* treatment are linked to the production of SCFAs, we analyzed SCFA levels in cecal samples obtained from naive, CIA, and CIA/*PG* mice. Our analysis revealed a significant reduction in acetate and propionate levels in CIA mice; however, *PG* administration did not restore the decreased levels of acetate and propionate induced by CIA (**Supplementary Figure 5**). No significant changes in butyrate levels were observed among the three experimental mouse groups.

The correlation analysis between pathobiological molecules and bacterial taxa revealed that significantly abundant bacterial taxa in the cecum of naïve mice tended to be positively correlated with ileal *Ocln* gene expression and negatively correlated with an inflammatory response (**Figure 6F**). Conversely, significantly abundant bacterial taxa in the cecum of CIA mice showed a tendency for a negative correlation with intestinal epithelial barrier function and a positive correlation with an inflammatory response (**Figure 6F**). The genus *Streptococcus* and species *S. danieliae* were discriminately enriched in the cecum of CIA/*PG* mice and were associated with improved intestinal barrier integrity, showing a positive correlation with ileal *Zo-1* gene expression and an inverse correlation with serum zonulin level (**Figure 6F**). Moreover, *Streptococcus* and *S. danieliae* were inversely correlated with the number of MLN Ly6C^+^ inflammatory monocytes and the gene expression of pro-inflammatory cytokines, such as *Ifng* and *Il17a*. Collectively, these findings demonstrated that the enriched microorganisms in the cecum of CIA/*PG* mice were closely linked to the amelioration of elevated gut permeability and inflammation.

## 4 Discussion

In this study, we identified altered antibodies against 36 microbial species in patients with RA compared to healthy controls using a serum IgM antibody microarray against 384 microbial species. Among them, *PG* was selected as a candidate microorganism likely to have a therapeutic effect among the bacterial species that were decreased in RA patients and displayed a negative correlation with disease activity. *PG* showed therapeutic potential due to its competitive growth with *P. gingivalis*, its secretion of immunomodulatory SCFAs, and its suppression of inflammatory cytokines associated with macrophage and T cell activation. In a mouse model of CIA, *PG* administration exerted a strong inhibitory effect on Th17 cells in ILN and markedly inhibited inflammatory monocytes in MLN and ILN. Moreover, *PG* improved gut permeability and tight junction disassembly, inhibiting intestinal inflammation in CIA mice.

The most common method currently used for gut microbiota identification is 16S rRNA sequencing of fecal samples. As previously highlighted, some studies have attempted to overcome the limitations of sequencing methods using feces by focusing on serum-based approaches. Continuous exposure to microbes can trigger an antibody response against these microorganisms, offering a potential metric for assessing microbial exposure (41). Although IgA has traditionally been associated with immune responses against gut microbiota, recent research has highlighted the significance of IgM and IgG in host-microbiota interactions (42). A recent study revealed the presence of numerous IgM^+^ B cells in human intestine, which produce secretory IgM and intestinal IgM reacting to a variety of microbiota (43). IgM, the primary antibody produced by B cells upon antigen exposure before class switching to IgG or IgA (44), is characterized by its large pentameric structure, enabling it to bind to a broader range of bacterial antigens compared to IgG and IgA (45). Consequently, IgM-associated alterations are likely to signify diverse and robust systemic immune responses during the initial stages of disease. This may effectively mirror recent or ongoing shifts in microbiota, serving as a first responder against microbial antigens.

Several studies have explored the potential role of IgM in the immunopathogenesis of various diseases, often mediated through altered gut microbiota. For instance, individuals with obesity have shown elevated concentrations of plasma IgM (46) along with an increase in IgM-bound gut microbiota (47). Patients with RA exhibited higher IgM and IgA levels compared to control (48), while most patients with poor prognosis displayed elevated levels of IgM antibodies specifically binding to IgG (49). Moreover, RA patients exhibited significantly higher levels of multiple antimicrobial response factors such as EndoCAb IgM and EndoCAb IgA, which are released into circulation in response to microbial exposure when compared to healthy individuals (48). Elevated levels of IgM specific to certain bacterial species were also observed in RA patients (50). Notably, sera from rheumatoid factor (RF)-positive individuals showed significantly higher levels of IgM antibodies, but not IgG and to a lesser extent IgA, against *Proteus mirabilis*, *Escherichia coli*, and *Klebsiella pneumoniae*, which have been linked with RA (51).

This study was conducted to analyze the microbiota of patients with RA using a serum microbial array designed to detect IgM antibodies against gut microbiota in the serum, building upon the rationale supported by prior research. Our results using this novel method revealed a reduction in the evenness of anti-microbial antibody abundances in patients with RA compared to controls, as well as distinct compositions in these antibody levels between the two groups. Antibodies against a total of 36 microbial species were differentially expressed, with 21 being decreased and 15 being increased in patients with RA compared to HC patients. Notably, we found a significant correlation between the anti-*PG* antibody and RA disease activity, suggesting the potential effectiveness of the RA-specific bacteria, *PG*, as a treatment avenue for RA.

Preliminary data and the literature accentuate the plausibility of employing *PG* for RA treatment. First, *PG* belongs to the microbial taxa associated with disease-suppressive soils (37). Second, *PG* produces butyrate and acetate, immunomodulatory metabolites derived from microbiota (38). Butyrate has been associated with improved proliferation of intestinal epithelial cells, mucin synthesis, and maintenance of mucosal immune homeostasis (52). Acetate is a major SCFA that can be converted to butyrate by gut microbial enzymes (53, 54). Similarly, we showed that *PG* produces butyrate and acetate *in-vitro*, indicating its potential to restore commensal bacterial composition. In addition, the growth of *PG* was inhibited in the presence of *P. gingivalis*, a recognized pathogenic player in RA. Although the specific bacteria involved in competitive interactions with *P. gingivalis* are unknown, *PG* appears to play a role in such interactions. Notably, *PG* administration partially restored certain genera and species, contributing to the partial recovery of gut microbiota homeostasis. This study revealed that *PG* could potentially improve CIA by reinstating eubiosis.

This study has limitations in determining whether *PG* exerts its arthritis-suppressing effects through SCFA production. While our *in-vitro* experiment confirmed *PG*’s capacity to generate SCFAs, we observed no significant differences in cecal SCFA levels between non-treated CIA and *PG*-treated CIA mice. A prior study demonstrated differences in SCFA levels between gut and serum, suggesting the potential systemic transport of SCFAs within the body (55). Their research revealed an increase in serum butyrate levels upon administering *Faeclibacterium prausnitzii*, a representative butyrate-producing bacterium, in CIA mice. However, they reported no distinctions in cecal butyrate levels between non-treated CIA and *F. prausnitzii*-treated CIA mice. Although we were unable to analyze serum SCFAs in our present study, future investigations may be necessary to explore the influence of *PG* on the redistribution of SCFAs between the gut and the circulatory system *in- vivo*.

Pathophysiological immune mechanisms between gut microbiota and RA pathogenesis are potentially multifactorial. Some of these potential interactions might be underlined by the activation of antigen-presenting cells (APCs) via toll-like receptors, enzymatic peptide citrullination induction, antigenic mimicry, control of the host immune system (triggering T cell differentiation), and increased Th17-mediated mucosal inflammation (56)*. P gingivalis* contributes to RA pathogenesis by activating Th17 cell-mediated pro-inflammatory responses and encoding peptidyl arginine deiminase, which facilitates the generation of ACPA (10, 11). Additionally, *P. copri* activates APCs through toll-like receptors and stimulates Th17 cell responses and ACPA production by B cells in patients with RA (56). Conversely, *P. histicola* can suppress arthritis development by modulating the immune response (regulation of DCs and generation of Treg cells), resulting in the suppression of Th17 responses and reduction of inflammatory cytokines (15). *PG* suppressed Th17 responses, as well as pro-inflammatory cytokines, inflammatory monocytes, and ACPA production. Our study further elucidates how *PG* contributes to CIA alleviation by curbing Th17 responses and inflammatory monocyte recruitment.

Lymphatic capillaries located within intestinal villi play a vital role in both nutrient absorption and immune cell trafficking to the MLN (57). Gut injuries can result in the release of pro-inflammatory factors that disrupt the integrity of the intestinal barrier (58). Recent evidence underscores the mesenteric lymph as a conduit for gut-derived harmful agents, ultimately contributing to systemic inflammation. These agents, upon absorption into the mesenteric lymphatic duct, possess the potential to activate and transport immune cells including monocytes/macrophages and DCs, thereby propagating systemic inflammation. During tissue injury or infection, monocytes rapidly infiltrate the affected tissues, acquiring pro-inflammatory characteristics, often classified as inflammatory monocytes (59). In inflamed colons, the recruitment of inflammatory monocytes to the LP is facilitated through CCR2, followed by their subsequent migration to the MLN, indicating colon inflammation (60).

In our study, although we did not observe a significant reduction in the population of inflammatory monocytes within the cLP of *PG*-treated CIA mice, we did find compelling evidence of *PG*’s inhibitory effect on the activation/recruitment of inflammatory monocytes within the MLN, implying its potential protective role against gut inflammation and injury. Furthermore, our study demonstrated a down-regulation of pro-inflammatory cytokine genes in the intestines of *PG*-treated CIA mice, accompanied by reduced serum zonulin levels, suggesting that *PG*’s impact on arthritis may be mediated through the modulation of the intestinal microenvironment. We also noted a trend toward reduced CCL2 gene expression in the ankles of *PG*-treated CIA mice, indicating a potential role for *PG* in inhibiting the migration of inflammatory monocytes to the synovium. Monocyte recruitment to the synovium relies on the interaction between chemokine receptors CCR2 and CX3CR1 on the monocyte surface and their ligands CCL2 and CX3CL1, which are produced by fibroblast-like synoviocytes (61). This interaction governs the egress of circulating monocytes and their subsequent recruitment into the RA synovium (62, 63).

Recent studies have highlighted the role of monocytes as APCs capable of activating T cells. In particular, Ly6C^+^ monocytes play a crucial role in transporting and presenting antigens to cognate T cells in LNs (59, 64). In RA, cytokines produced by monocytes/macrophages, such as IL-1β, IL-6, and IL-23, are instrumental in driving the polarization of Th17 cells and enhancing IL-17 production in CD4^+^ T cells (65). Inflammatory monocytes are likely the predominant monocyte subset involved in modulating the Th17 cells response, contributing to the activation and expansion of Th17 cells in inflamed synovial tissue (66, 67). Based on the findings from previous research, it is reasonable to speculate that *PG* contributes to the reduction of inflammatory monocyte recruitment/infiltration in the MLN by mitigating gut inflammation and barrier disruption. Concurrently, *PG* may hinder the trafficking of inflammatory monocytes to the synovium and its draining ILN by regulating CCL2, thereby influencing the induction of Th17 cells in the synovium and preventing the dissemination of systemic inflammation.

Nonetheless, recent studies have unveiled the intricate role of monocytes, including their actions within circulation and migration into tissues and lymphoid organs (59). Monocytes precursors originating from the bone marrow (BM) migrate to diverse organs, including lymphoid tissues like LNs and spleen, as well as nonlymphoid tissues such as the intestines, lung, and skin through blood circulation (68). CCL2 also plays a critical role in the egress of Ly6C^hi^ monocytes from the BM (64). Activated monocytes migrate from the bloodstream into inflamed tissues through chemotaxis mediated CCL2 and CX3CL1, contributing to proinflammatory cytokine production. Monocytes can also traffic to LNs through tissues (69). Hence, it is conceivable that during arthritis, monocytes originating from the BM and circulating in the bloodstream may migrate to inflamed synovium or intestine, subsequently emigrating to their respective draining LNs. In the exploration of *PG*’s mechanisms for mitigating arthritis, unresolved questions remain regarding monocyte migration to tissues and draining LNs, as well as the interactions occurring within this lymphoid network.

Our findings indicate that *PG* influences intestinal permeability, suggesting a potential therapeutic role for *PG* in arthritis by modulating the intestinal environment. Zonulin, a central factor in this process, disrupts intestinal tight-junction proteins, enhances intestinal permeability, and promotes Th1 and Th17 infiltration in the lamina propria before the onset of arthritis (40). A recent study demonstrated that oral treatment with the zonulin antagonist larazotide before the onset of arthritis prevented increased intestinal barrier permeability and attenuated arthritis symptoms in CIA mice (40). ZO-1, an intracellular adaptor protein that binds to numerous transmembrane and cytoplasmic proteins, is required for the assembly of both adherens and tight junctions (70). Our study demonstrated that *PG* supplementation restored intestinal barrier integrity, decreased serum levels of zonulin, and increased gene expression of intestinal tight junction molecules (*Zo-1* and *Ocln*), while also suppressing pro-inflammatory cytokines in the intestine. Thus, *PG* has the potential to ameliorate CIA by restoring intestinal barrier function. However, further mechanistic study is needed to determine which components of *PG*, including its structure or derived metabolites, contribute to the enhancement of intestinal function *in vivo*.

In the current investigation, certain limitations warrant acknowledgment. We did not compare the serum microbial array results with those obtained through fecal 16S RNA sequencing, the most common method for microbiota identification. Furthermore, since the serum microbial arrays examined only 384 microbial strains, the interpretation of the results could be somewhat limited. Lastly, the potential influence of anti-rheumatic drugs on microbiota was not factored into our analysis.

In conclusion, this study reiterates the utility of serum anti-microbial antibody arrays in uncovering therapeutic microbial targets for RA. Furthermore, we demonstrate the therapeutic effect of *PG* in restoring the intestinal barrier integrity and suppressing the immune response against RA. Therefore, *PG* may represent a promising microbiota-targeted therapy for individuals with RA.

## Data availability statement

The data presented in the study are deposited in the NCBI Sequence Read Archive (SRA) repository, accession number PRJNA1048658 (https://www.ncbi.nlm.nih.gov/sra/PRJNA1048658).

## Ethics statement

The studies involving humans were approved by GNUH Institutional Review Board in Korea (permit No: GNUH 2017-08-015). The studies were conducted in accordance with the local legislation and institutional requirements. The participants provided their written informed consent to participate in this study. The animal study was approved by the Institutional Animal Care and Use Committee of GNU in Korea (IACUC approval ID: GNU-200724-M0046). The study was conducted in accordance with the local legislation and institutional requirements.

## Author contributions

SK: Data curation, Formal analysis, Funding acquisition, Methodology, Visualization, Writing – original draft, Writing – review & editing. SC: Data curation, Formal analysis, Methodology, Visualization, Writing – original draft, Writing – review & editing. YC: Writing – review & editing. MK: Writing – review & editing. HK: Writing – review & editing. HL: Data curation, Formal analysis, Writing – review & editing. SH: Data curation, Formal analysis, Methodology, Resources, Writing – original draft, Conceptualization, Writing – review & editing. SP: Methodology, Resources, Writing – review & editing. MP: Methodology, Resources, Writing – review & editing. YS: Data curation, Formal analysis, Funding acquisition, Methodology, Supervision, Writing – original draft, Writing – review & editing, Conceptualization. SL: Data curation, Formal analysis, Funding acquisition, Methodology, Supervision, Writing – original draft, Writing – review & editing, Conceptualization.
