# Supplementary material for: Peptoniphilus gorbachii alleviates collagen-induced arthritis in mice by improving intestinal homeostasis and immune regulation
Source: Front Immunol. 2024 Jan 4;14:1286387. doi: 10.3389/fimmu.2023.1286387 (PMC10794505; doi:10.3389/fimmu.2023.1286387)
Supplement: Supplementary file 1 [file DataSheet_1.docx]

Supplementary Material

*Peptoniphilus gorbachii* alleviates collagen-induced arthritis in mice by improving intestinal homeostasis and immune regulation

Suhee Kim^1†^, Sung Hak Chun^1†^, Yun-Hong Cheon^1^, Mingyo Kim^1^, Hyun-Ok Kim^1,2^, Hanna Lee^1,2^, Seong-Tshool Hong^3^, Sang-Jun Park^4^, Myeong Soo Park^4^, Young Sun Suh^1,2*^ and Sang-Il Lee^1*^

*** Correspondence.** Young Sun Suh: [tatabox123@hanmail.net](mailto:tatabox123@hanmail.net); Sang-Il Lee: [goldgu@gnu.ac.kr](mailto:goldgu@gnu.ac.kr)

**Supplementary Table 1.** Clinical information for healthy individuals and patients with RA

| **Parameters** | **Healthy**  **(*n* = 50)** | **RA (*n* = 81)** | | | ***p* value** |
| --- | --- | --- | --- | --- | --- |
|  |  | LoDA  (*n* = 30) | MHDA  (*n* = 51) | Total  (*n* = 81) |  |
| **Age, years, mean (median)** | 60.5 (60.0) | 61.0 (61.0) | 59.9 (61.0) | 60.3 (61.0) | 0.83^a^ |
| **Female, %** | 68.0% | 70.0% | 66.7% | 67.9% | 0.95^a^ |
| **Disease activity parameters**  ESR, mm/h, mean (median)  CRP, mg/dL, mean (median)  TJC-28, mean (median)  SJC-28, mean (median)  Pain VAS  DAS28-ESR, mean (median)  DAS28-CRP, mean (median) | –  –  –  –  –  –  – | 19.9 (14.0)  1.1 (0.7)  0.9 (1.0)  0.3 (0.0)  21.8 (20.0)  2.5 (2.6)  2.0 (2.0) | 53.5 (45.0)  9.4 (4.2)  3.8 (2.0)  2.8 (2.0)  43.9 (40.0)  4.6 (4.3)  3.6 (3.3) | 41.1 (36.0)  6.3 (2.2)  2.7 (2.0)  1.8 (1.0)  35.7 (30.0)  3.9 (3.6)  3.0 (2.8) | < 0.01^b^  < 0.01^b^  < 0.01^b^  < 0.01^b^  < 0.01^b^  < 0.01^b^  < 0.01^b^ |

ESR: Erythrocyte Sedimentation Rate, CRP: C-Reactive Protein, TJC: 28 Tender Joint Count, SJC: 28 Swollen Joint Count, VAS: Visual Analog Scale, DAS: Disease Activity Score, LoDA: Low disease activity (DAS28-ESR <3.2), MHDA: Moderate to high disease activity (DAS28-ESR ≥ 3.2). ^a^*p* > 0.05 among healthy, LoDA, and MHDA groups, ^b^*p* < 0.01 between LoDA and MHDA groups.

**Supplementary Table 2.** Microbial species used for antibody microarray

| **Phylum** | **Microbial species** |
| --- | --- |
| **Actinobacteria** | *Collinsella intestinalis, Collinsella stercoris, Collinsella tanakaei, Collinsella massiliensis, Collinsella aerofaciens, Enorma timonensis, Atopobium rimae, Olsenella uli, Streptomyces sp, Microbacterium laevaniformans, Actinomyces viscosus, Actinomyces israelii, Actinomyces odontolyticus, Bifidobacterium pseudolongum, Bifidobacterium breve, Bifidobacterium infantis, Bifidobacterium scardovii, Bifidobacterium longum, Bifidobacterium dentium, Bifidobacterium adolescentis, Bifidobacterium catenulatum, Bifidobacterium pseudocatenulatum, Gardnerella vaginalis, Bifidobacterium choerinum, Parascardovia denticolens, Mycobacterium smegmatis, Gordonia aichiensis, Corynebacterium amycolatum, Corynebacterium propinquum, Corynebacterium singulare, Corynebacterium imitans, Corynebacterium diphtheriae, Corynebacterium pilbarense, Corynebacterium kroppenstedtii, Micrococcus lylae, Micrococcus sp, Kocuria kristinae, Arthrobacter oxydans, Brevibacterium epidermidis, Rothia endophytica, Propionibacterium granulosum, Propionibacterium acnes, Propionibacterium avidum, Luteococcus peritonei, Gordonibacter urolithinfaciens, Slackia isoflavoniconvertens* |
| **Bacteroidetes** | *Bacteroides finegoldii, Bacteroides faecis JCM 16477, Bacteroides fragilis, Bacteroides coprocola, Bacteroides koreensis YS-aM39, Porphyromonas gingivalis, Porphyromonas bennonis, Parabacteroides distasonis, Prevotella micans, Alistipes indistinctus* |
| **Firmicutes** | *Bacillus alcalophilus, Bacillus cereus, Bacillus amyloliquefaciens, Bacillus megaterium, Bacillus pumilus, Bacillus marisflavi, Bacillus firmus, Bacillus clausii, Bacillus niacini, Bacillus novalis, Bacillus ciccensis, Bacillus safensis, Bacillus thermoamylovorans, Bacillus subtilis, Bacillus sp, Bacillus mycoides, Bacillus atrophaeus, Bacillus sonorensis, Bacillus thuringiensis, Bacillus drentensis, Bacillus flexus, Bacillus altitudinis, Bacillus indicus, Bacillus aquimaris, Bacillus nealsonii, Bacillus velezensis, Bacillus massiliogorillae, Bacillus wiedmannii, Bacillus zanthoxyli, Exiguobacterium acetylicum, Exiguobacterium undae, Lysinibacillus macroides, Lysinibacillus xylanilyticus, Terribacillus goriensis, Listeria monocytogenes, Aneurinibacillus aneurinolyticus, Paenibacillus barengoltzii, Paenibacillus lautus, Paenibacillus tundrae, Paenibacillus sp, Paenibacillus glucanolyticus, Paenibacillus dendritiformis, Paeniclostridium ghonii, Paraclostridium bifermentans subsp. muricolitidis, Sporosarcina koreensis, Staphylococcus lugdunensis, Staphylococcus simulans, Staphylococcus lentus, Staphylococcus hominis, Staphylococcus auricularis, Staphylococcus warneri, Staphylococcus epidermidis, Staphylococcus capitis subsp. capitis, Staphylococcus capitis subsp. Urealyticus, Staphylococcus sciuri subsp. sciuri, Staphylococcus arlettae, Staphylococcus chromogenes, Staphylococcus vitulinus, Staphylococcus kloosii, Staphylococcus succinus subsp. casei, Staphylococcus equorum, Staphylococcus Nepalensis, Staphylococcus cohnii, Staphylococcus xylosus, Staphylococcus aureus, Staphylococcus pasteuri, Staphylococcus argenteus, Abiotrophia defectiva, Aerococcus sanquinicola, Aerococcus viridans , Granulicatella adiacens, Enterococcus malodoratus, Enterococcus gallinarum, Enterococcus caccae, Enterococcus faecalis, Enterococcus durans, Enterococcus hirae, Enterococcus casseliflavus, Enterococcus pallens, Enterococcus avium, Enterococcus raffinosus, Enterococcus gilvus, Enterococcus faecium, Vagococcus fluvialis, Lactobacillus crispatus, Lactobacillus gallinarum, Lactobacillus acidophilus, Lactobacillus ultunensis, Lactobacillus intestinalis, Lactobacillus kalixensis, Lactobacillus jensenii, Lactobacillus iners, Lactobacillus johnsonii, Lactobacillus gasseri, Lactobacillus gastricus, Lactobacillus fermentum, Lactobacillus reuteri, Lactobacillus vaginalis, Lactobacillus oris, Lactobacillus antri, Lactobacillus amylotrophicus, Lactobacillus bifermentans, Lactobacillus dextrinicus, Lactobacillus farciminis, Lactobacillus alimentarius, Lactobacillus collinoides, Lactobacillus plantarum, Lactobacillus brevis, Lactobacillus parabuchneri, Lactobacillus kefiri, Lactobacillus buchneri, Lactobacillus lindneri, Lactobacillus fructivorans, Lactobacillus graminis, Lactobacillus coryniformis, Lactobacillus pantheri, Lactobacillus rhamnosus, Lactobacillus zeae, Lactobacillus mali, Lactobacillus saerimneri, Lactobacillus salivarius, Lactobacillus agilis, Lactobacillus ruminis, Lactobacillus sp, Lactobacillus murinus, Lactobacillus taiwanensis, Lactobacillus amylovorus, Lactobacillus paracasei HBUAS51140, Lactobacillus pentosus, Lactobacillus delbrueckii subsp. lactis, Lactobacillus delbrueckii subsp. delbrueckii, Lactobacillus animalis, Lactobacillus curvatus, Lactobacillus mucosae, Lactobacillus sharpeae, Lactobacillus kitasatonis, Pediococcus acidilactici, Pediococcus pentosaceus, Leuconostoc lactis KCCM202369, Lactococcus sp. XJ133-127-4NG1, Leuconostoc citreum JCM 9698, Leuconostoc mesenteroides A1, Leuconostoc pseudomesenteroides, Leuconostoc sp. F1, Weissella sp. IMAU50209, Weissella confusa qz-484, Weissella cibaria CH3, Lactococcus lactis, Lactococcus garvieae, Okadaella gastrococcus, Streptococcus sobrinus, Streptococcus mutans, Streptococcus anginosus, Streptococcus constellatus, Streptococcus sanguis, Streptococcus salivarius, Streptococcus thermophilus, Streptococcus vestibularis, Streptococcus pyogenes, Streptococcus pasteuri, Streptococcus lutetiensis, Streptococcus infantis, Streptococcus oralis, Streptococcus pneumoniae, Streptococcus peroris, Streptococcus parasanguis, Streptococcus gordonii, Streptococcus cristatus, Streptococcus australis, Streptococcus sanguinis, Streptococcus agalactiae XMF12, Streptococcus dysgalactiae subsp. equisimilis CIP 105120, Streptococcus alactolyticus, Streptococcus gallolyticus, Streptococcus equi subsp. equi, Streptococcus equinus , Streptococcus thoraltensis, Streptococcus hyointestinalis, Streptococcus ratti, Streptococcus intermedius, Peptococcus niger, Clostridium baratti, Clostridium aldenense, Clostridium butyricum, Clostridium celatum, Clostridium innocuum, Clostridium colicanis, Clostridium methylpentosum, Clostridium sphenoides, Clostridium scindens, Clostridium nexile, Clostridium sp, Clostridium hydrogeniformans, Dorea longicatena, Eubacterium rectale, Eubacterium saburreum, Blautia faecis, Blautia hansenii, Catenibacterium mitsuokai, Clostridium cochlearium, Clostridium indolis, Clostridium sartagoforme, Eubacterium limosum, Johnsonella ignava, Lachnospira multipara, Roseburia hominis, Stomatobaculum longum, Romboutsia lituseburensis, Anaerotruncus colihominis, Faecalibacterium prausnitzii, Ruminococcus gnavus, Terrisporobacter glycolicus, Coprobacillus catenaformis, Bulleidia extructa, Solobacterium moorei, Dielma fastidiosa, Eubacterium cylindroides, Dialister pneumosintes, Veillonella parvula, Veillonella denticariosi, Peptoniphilus gorbachii, Peptoniphilus grossensis, Peptoniphilus sp, Peptoniphilus koenoeneniae, Peptoniphilus asaccharolyticus, Peptoniphilus ivoricus, Anaerococcus hydrogenalis, Anaerococcus senegalensis, Anaerococcus prevotii, Anaerococcus tetradius* |
| **Flavobacteria** | *Capnocytophaga granulosa, Capnocytophaga ochracea* |
| **Fusobacteria** | *Fusobacterium varium, Cetobacterium somerae* |
| **Proteobacteria** | *Sphingomonas paucimobilis, Sphingomonas adhaesiva, Ochrobactrum cytisi, Ochrobactrum anthropi, Enhydrobacter aerosaccus, Neisseria mucosa, Neisseria gonorrhoeae, Neisseria meningitidis, Achromobacter denitrificans, Alcaligenes faecalis, Achromobacter xylosoxidans, Ralstonia pickettii, Pandoraea pnomenusa, Pandoraea sputorum, Ralstonia mannitolilytica, Ralstonia sp, Comamonas kerstersii, Sutterella wadsworthensis , Campylobacter sputorum, Campylobacter showae, Buttiauxella gaviniae, Buttiauxella agrestis, Citrobacter sedlakii, Citrobacter werkmanii, Citrobacter freundii, Cronobacter sakazakii, Edwardsiella tarda, Enterobacter cloacae, Enterobacter hormaechei, Escherichia vulneris, Escherichia coli, Escherichia coli O157, Escherichia fergusonii, Ewingella americana, Klebsiella pneumoniae, Klebsiella sp. SCAUS56, Klebsiella variicola ALK036, Kocuria marina, Klebsiella oxytoca, Leclercia adecarboxylata, Proteus vulgaris, Proteus penneri, Raoultella ornithinolytica, Raoultella sp, Salmonella enterica, Salmonella ParatyphiA, Salmonella Typhimurium, Shigella boydii, Shigella sonnei, Yersinia kristensenii, Morganella morganii MS6, Serratia marcescens, Serratia liquefaciens, Serratia ficaria, Aeromonas rivipollensis, Aeromonas hydrophila, Aeromonas allosaccharophila, Aeromonas caviae, Aeromonas enteropelogenes, Aeromonas veronii, Pseudomonas aeruginosa, Psychrobacter phenylpyruvicus, Acinetobacter calcoaceticus, Acinetobacter baumannii, Acinetobacter pittii, Acinetobacter sp, Acinetobacter variabilis, Moraxella osloensis, Pseudomonas putida, Pseudomonas sp, Pseudomonas fluorescens, Pseudomonas guariconensis, Stenotrophomonas pavanii, Stenotrophomonas sp, Stenotrophomonas maltophilia* |
| **Tenericutes** | *Mycoplasma lipophilum* |
| **Ascomycota** | *Atopobium parvulum, Aspergillus niger, Aspergillus fumigatus, Aspergillus sydowii, Saccharomyces cerevisiae, Candida rugosa, Candida albicans, Candida tropicalis* |
| **Basidiomycota** | *Trichosporon inkin, Cryptococcus diffluens, Cryptococcus neoformans* |
| **Zygomycota** | *Rhizopus oryze* |
| **Viridiplantae** | *Triticum aestivum* |
| **Unclassified** | *Bacterium QHB34, Bacterium NLAE-zl-H211, Uncultured_1, 3, and 4* |

**Supplementary Table 3.** Primer sequences for quantitative real-time PCR

| **Species** | **Gene symbol** | **Direction** | **Sequence** |
| --- | --- | --- | --- |
| Mouse | *Il6* | Forward  Reverse | 5’- GGCCTTCCCTACTTCACAAG -3’  5’- ATTTCCACGATTTCCCAGAG -3’ |
| Mouse | *Il1b* | Forward  Reverse | 5’- CTGGTGTGTGACGTTCCCATTA -3’  5’- CCGACAGCACGAGGCTTT -3’ |
| Mouse | *Il23* | Forward  Reverse | 5’- ATGCCCAGCCTGAGTTCTAGTC -3’  5’- GAGGCTTCGAAGGATCTTGGA -3’ |
| Mouse | *Tnfa* | Forward  Reverse | 5’- GACCCTCACACTCAGATCATCT -3’  5’- CCTCCACTTGGTGGTTTGCT -3’ |
| Mouse | *Il10* | Forward  Reverse | 5’- GCTCTTACTGACTGGCATGAG -3’  5’- CGCAGCTCTAGGAGCATGTG -3’ |
| Mouse | *Ifng* | Forward  Reverse | 5’- TCTTCTTGGATATCTGGAGGAACTG -3’  5’- GAGATAATCTGGCTCTGCAGGATT -3’ |
| Mouse | *Il17a* | Forward  Reverse | 5’- CCGCAATGAAGACCCTGATAG -3’  5’- TCCCTCCGCATTGACACA -3’ |
| Mouse | *Cx3cr1* | Forward  Reverse | 5’- CACCATTAGTCTGGGCGTCT -3’  5’- GATGCGGAAGTAGCAAAAGC -3’ |
| Mouse | *Ccl2* | Forward  Reverse | 5’- CCAGCCTACTCATTGGGAT -3’  5’- GGGCCTGCTGTTCACAGTT -3’ |
| Mouse | *Zo-1* | Forward  Reverse | 5’- CCTCCGTTGCCCTCACAGTA -3’  5’- CCCTTGGAATGTATGTGGAGAGA -3’ |
| Mouse | *Ocln* | Forward  Reverse | 5’- GTCCACCTCCTTACAGACCTGATG -3’  5’- TCGGCCGGACATGCAT -3’ |
| Mouse | *Gapdh* | Forward  Reverse | 5’- ACCCAGAAGACTGTGGATGG -3’  5’- ACACATTGGGGGTAGGAACA -3’ |
| Mouse | *Hprt* | Forward  Reverse | 5’- TGTGGCCATCTGCCTAGTAAAG -3’  5’- GGCTCATAGTGCAAATCAAAAGTC -3’ |

**Supplementary Table 4.** Cell staining antibodies for flow cytometry

| **Antibodies** | **Source** |
| --- | --- |
| FVS510 | BD Bioscience |
| Anti-mouse CD4, PE/Cy7, clone GK1.5 | eBioscience |
| Anti-mouse IFN-γ, FITC, clone XMG1.2 | eBioscience |
| Anti-mouse IL-17A, APC, clone eBio17B7 | eBioscience |
| Anti-mouse Foxp3, APC, clone FJK-16s | eBioscience |
| Anti-mouse CD45, APC/Cy7, clone 30-F11 | BD Bioscience |
| Anti-mouse CD64, BV780, clone X54-5/7.1 | BD Bioscience |
| Anti-mouse CD11b, BB515, clone M1/70 | BD Bioscience |
| Anti-mouse CX3CR1, APC, clone SA011F11 | BioLegend |
| Anti-mouse MHCII (I-A/I-E), BV421, clone M5/114.15.2 | BioLegend |
| Anti-mouse Ly-6C, BV605, clone AL-21 | BD Bioscience |
| Anti-mouse CD11c, BB700, clone HL3 | BD Bioscience |
| Anti-mouse CD103, PE, clone 2E7 | BioLegend |

**Supplementary Table 5.** *Peptoniphilus gorbachii* as a candidate for treating RA – literature review

| **Microbial candidates** | **Microbial array findings in this study** | **Published source** |
| --- | --- | --- |
| *Ochrobactrum* (O.) *cytisi* | Decreased abundance in patients with RA  Inverse association with disease severity  A co-occurrence network with antibodies against other potential candidates, *P. gorbachii* and *D. pneumosintes*, in patients with RA | *O. cytisi* strains, Pv2Z2 and IPA7.2, promotes growth of plants (1, 2) |
| *Peptoniphilus* (P.) *gorbachii* | Decreased abundance in patients with RA  Inverse association with disease severity  A co-occurrence network with antibodies against other potential candidates, *O. cytisi* and *D. pneumosintes*, in patients with RA  In patients with RA, shows an inverse correlation with anti-*P. gingivalis* antibody, a putative pathogen implicated in RA | Protective potential against infection by soil-borne pathogen (3, 4)  Genus *Peptoniphilus* comprises butyrate-producing species (5) |
| *Dialister* (D.) *pneumosintes* | Decreased abundance in patients with RA  Inverse association with disease severity  A co-occurrence network with antibodies against other potential candidates, *O. cytisi* and *P. gorbachii*, in patients with RA | Suspected periodontal pathogen (6) |
| *Veillonella* (V.) *denticariosi* | Decreased abundance in patients with RA  Inverse association with disease severity | Enriched in carious dentine (7)  *Veillonella* species play an important role in human oral microbiome and may have dual role; potential accessory pathogens or anti-cariogenic activity (8) |


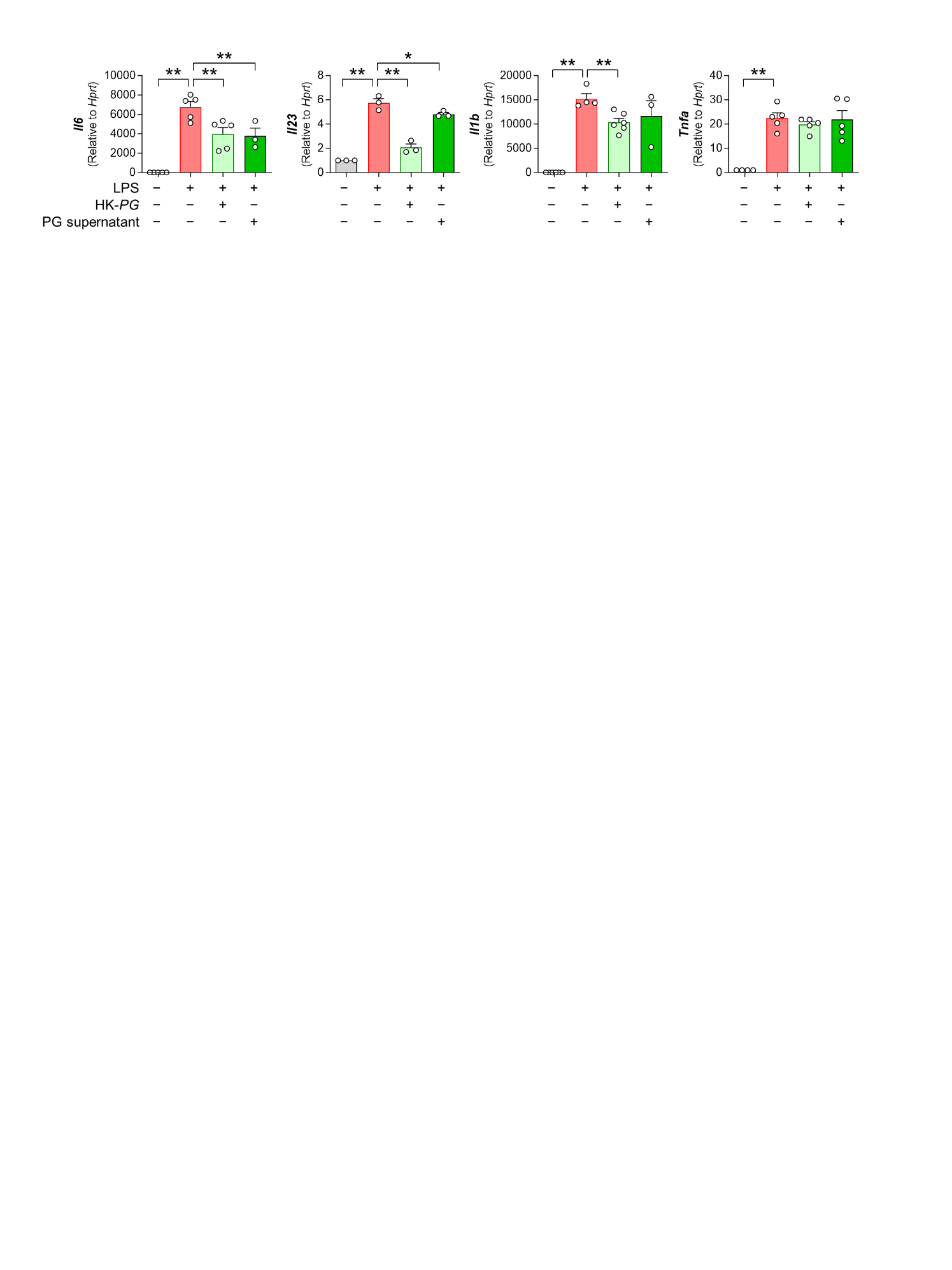


Supplementary Figure 1. Pro-inflammatory gene expression of RAW 264.7 cells following treatment with HK-*PG* or *PG* supernatant under LPS stimulation. Cells were exposed to HK-*PG* (multiplicity of infection 1, MOI 1) or *PG* supernatant (0.5%) for 20 h, followed by stimulation with LPS for an additional 4 h. The expression of target genes relative to hypoxanthine-guanine phosphoribosyltransferase (*Hprt*) was then evaluated. *n* = 3–6 per group. Mean ± SEM. One-way ANOVA, Fisher’s LSD *post hoc* test, **p* < 0.05, ***p* < 0.01 vs LPS^+^ control.


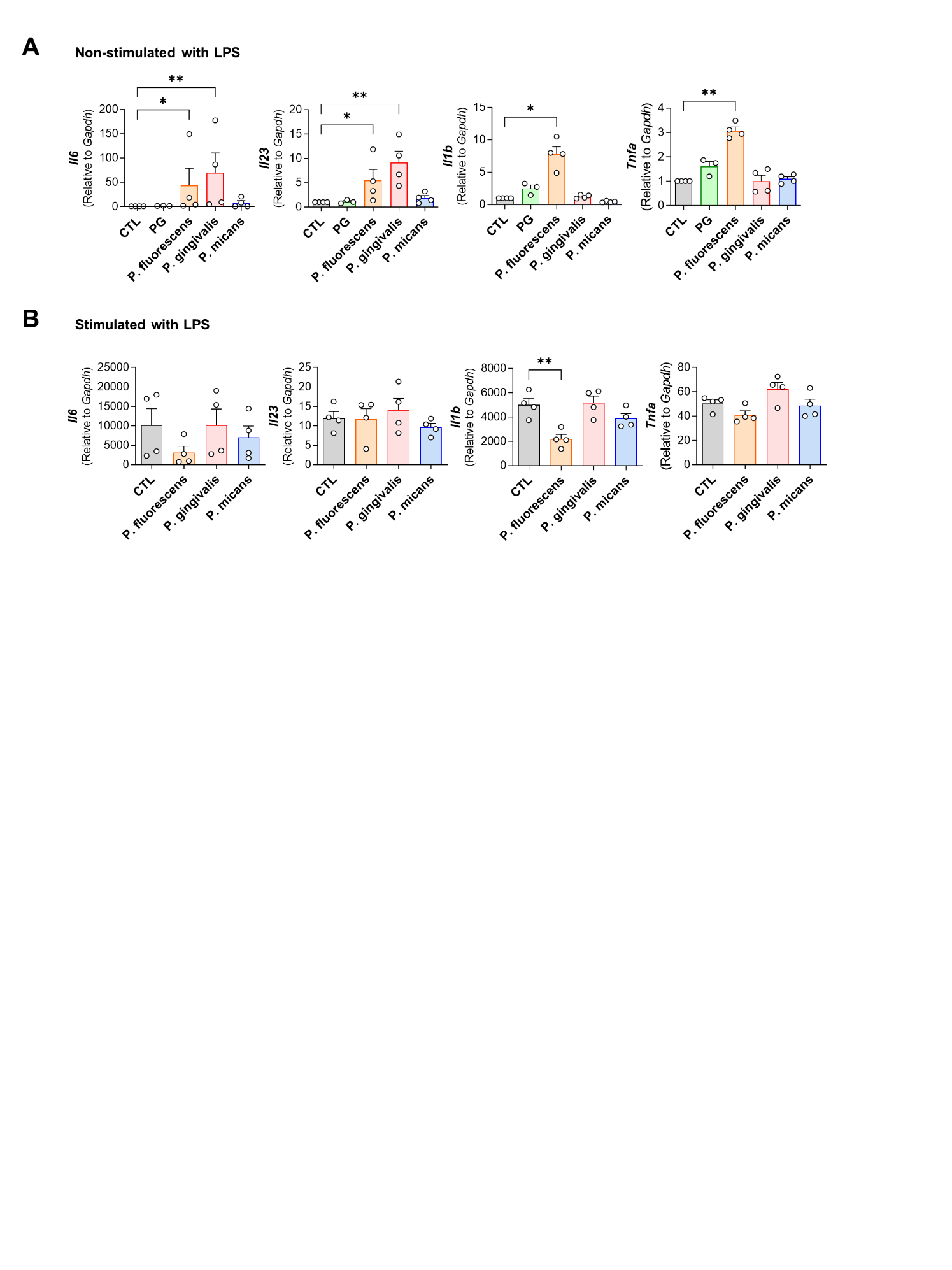


**Supplementary Figure 2.** The expression of pro-inflammatory cytokine genes in RAW 264.7 cells following treatment with various bacteria. RAW 264.7 cells were cultured in the absence (CTL) or presence of heat-killed (HK)-*PG*, *P. fluorescens*, *P. gingivalis*, or *P. micans*, under both absence **(A)** and presence of LPS **(B)** conditions. Cells were exposed to HK-bacteria (MOI 1) for 20 h, followed by stimulation with LPS for an additional 4 h. Subsequently, total RNA extraction from the cells was performed to assess pro-inflammatory gene expression. *n* = 3–4 per group. Mean ± SEM. Kruskal-Wallis, Dunn’s *post hoc* test, **p* < 0.05, ***p* < 0.01 vs CTL.


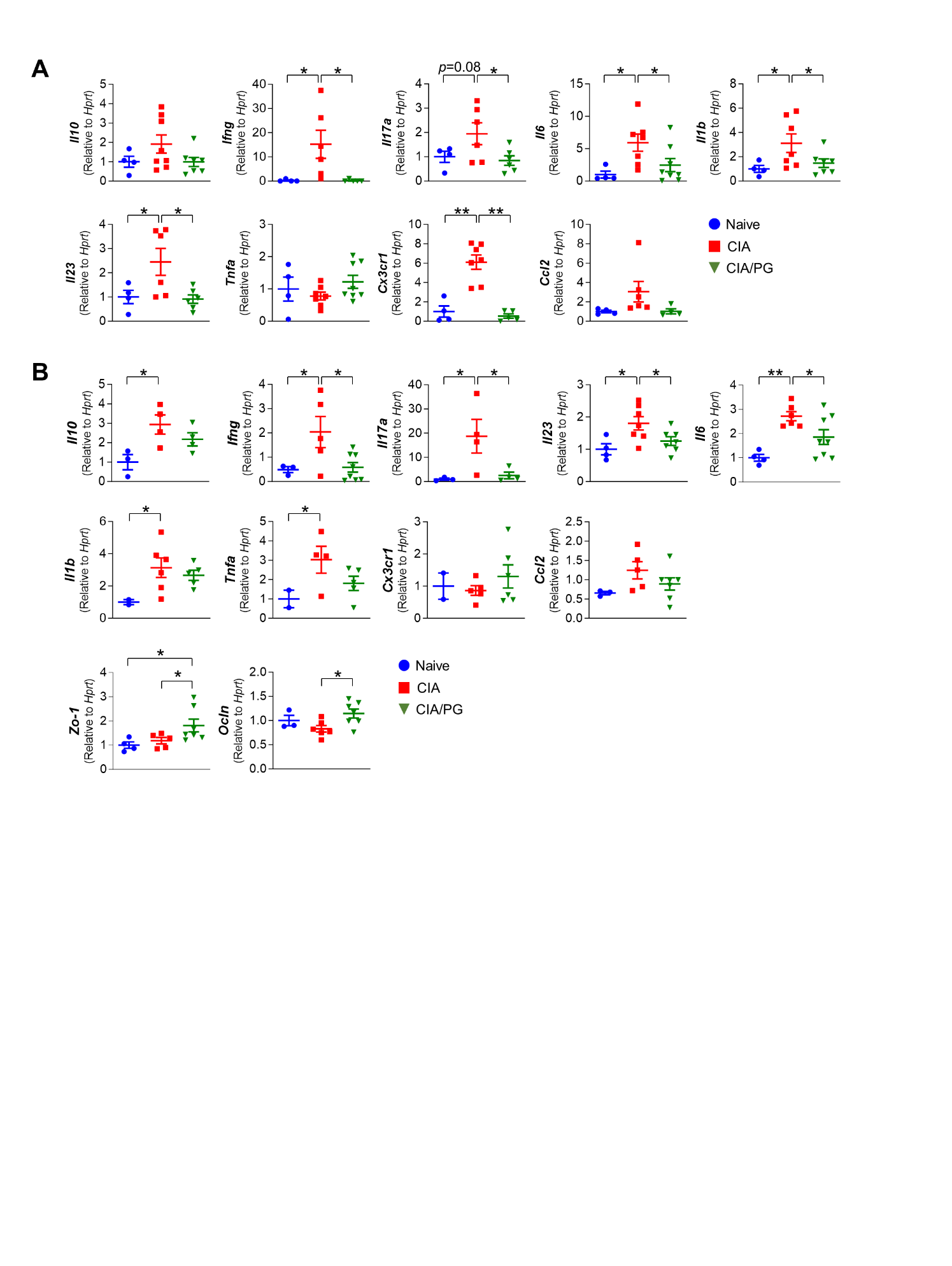


Supplementary Figure 3. Gene expression profiles relative to *Hprt* in ankle and ileum samples obtained from naïve (*n* = 2–4), CIA (*n* = 4–8), and CIA/*PG* (*n* = 4–8) mice. (A) The gene expression of pro-inflammatory cytokines in ankle samples. (B) The gene expression of pro-inflammatory cytokines and tight-junction molecules in ileum samples. Mean ± SEM. One-way ANOVA, Fisher’s LSD *post hoc* test, **p* < 0.05.


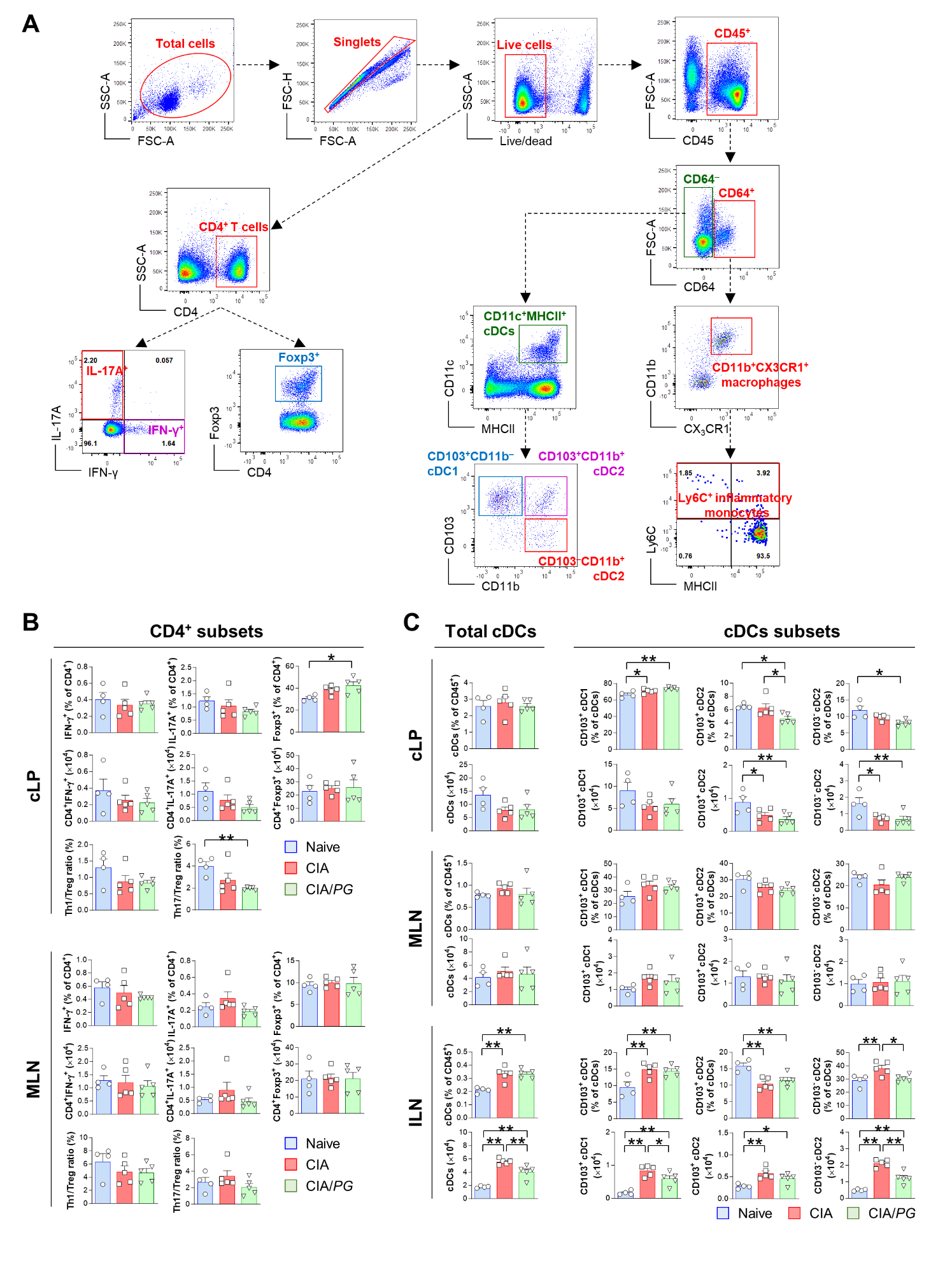


Supplementary Figure 4. Flow cytometric analysis of immune cell populations in naïve, CIA, and CIA/*PG* mice. (A) Flow cytometric gating strategy for CD4^+^ T cell subsets, conventional dendritic cells (cDCs), and macrophage lineages. (B) Flow cytometric analysis of CD4^+^ T cell and cDC subsets from cLP, MLN, and ILN of naïve (*n* = 4), CIA (*n* = 5), and CIA/*PG* (*n* = 5) mice. Mean ± SEM. One-way ANOVA, Fisher’s LSD *post hoc* test, **p* < 0.05.


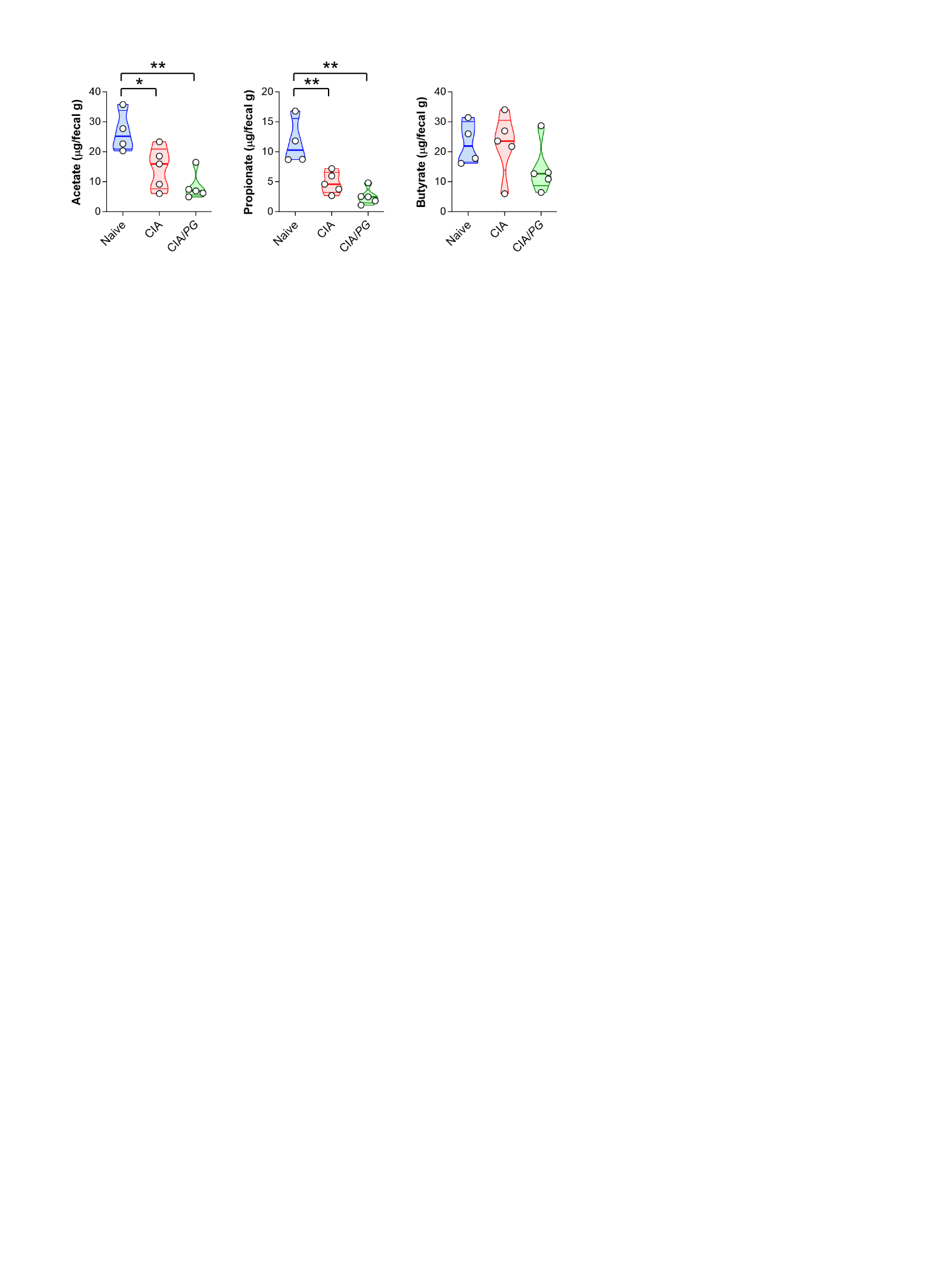


Supplementary Figure 5. Measurement of short-chain fatty acid (SCFA) levels in cecal samples obtained from naïve (*n* = 4), CIA (*n* = 5), and CIA/*PG* (*n* = 5) mice. Data are expressed as median and interquartile range (1^st^ – 3^rd^ quartile). One-way ANOVA, Fisher’s LSD *post hoc* test, **p* < 0.05, ***p* < 0.01.

**References**

1. Burygin GL, Kargapolova KY, Kryuchkova YV, Avdeeva ES, Gogoleva NE, Ponomaryova TS, et al. Ochrobactrum cytisi ipa7.2 promotes growth of potato microplants and is resistant to abiotic stress. *World J Microbiol Biotechnol* (2019) 35:55. doi: [10.1007/s11274-019-2633-x](https://doi.org/10.1007/s11274-019-2633-x).

2. Imran A, Saadalla MJA, Khan S-U, Mirza MS, Malik KA, Hafeez FY. Ochrobactrum sp. Pv2z2 exhibits multiple traits of plant growth promotion, biodegradation and n-acyl-homoserine-lactone quorum sensing. *Annals of microbiology* (2014) 64:1797-806.

3. Adam M, Westphal A, Hallmann J, Heuer H. Specific microbial attachment to root knot nematodes in suppressive soil. *Appl Environ Microbiol* (2014) 80:2679–86. doi: [10.1128/AEM.03905-13](https://doi.org/10.1128/aem.03905-13).

4. Gómez Expósito R, de Bruijn I, Postma J, Raaijmakers JM. Current insights into the role of rhizosphere bacteria in disease suppressive soils. *Front Microbiol* (2017) 8:2529. doi: [10.3389/fmicb.2017.02529](https://doi.org/10.3389/fmicb.2017.02529).

5. Murphy EC, Frick IM. Gram-positive anaerobic cocci--Commensals and opportunistic pathogens. *FEMS Microbiol Rev* (2013) 37:520–53. doi: [10.1111/1574-6976.12005](https://doi.org/10.1111/1574-6976.12005).

6. Kaiser M, Weis M, Kehr K, Varnholt V, Schroten H, Tenenbaum T. Severe pneumonia and sepsis caused by Dialister pneumosintes in an adolescent. *Pathogens* (2021) 10. doi: [10.3390/pathogens10060733](https://doi.org/10.3390/pathogens10060733).

7. Zhou P, Manoil D, Belibasakis GN, Kotsakis GA. Veillonellae: Beyond bridging species in oral biofilm ecology. *Front Oral Health* (2021) 2:774115. doi: [10.3389/froh.2021.774115](https://doi.org/10.3389/froh.2021.774115).

8. Mashima I, Liao YC, Lin CH, Nakazawa F, Haase EM, Kiyoura Y, et al. Comparative pan-genome analysis of oral Veillonella species. *Microorganisms* (2021) 9. doi: [10.3390/microorganisms9081775](https://doi.org/10.3390/microorganisms9081775).
